# Association of childhood smoking and adult mortality: prospective study of 120 000 Cuban adults

**DOI:** 10.1016/S2214-109X(20)30221-7

**Published:** 2020-05-21

**Authors:** Blake Thomson, Nurys Armas Rojas, Ben Lacey, Julie Ann Burrett, Patricia Varona-Pérez, Marcy Calderón Martínez, Elba Lorenzo-Vázquez, Sonia Bess Constantén, José Manuel Morales Rigau, Osvaldo Jesús Hernández López, Miguel Ángel Martínez Morales, Ismell Alonso Alomá, Fernando Achiong Estupiñan, Mayda Díaz González, Noel Rosquete Muñoz, Marelis Cendra Asencio, Jonathan Emberson, Richard Peto, Sarah Lewington, Alfredo Dueñas Herrera

**Affiliations:** a Clinical Trial Service Unit and Epidemiological Studies Unit, Nuffield Department of Population Health, University of Oxford, Oxford, UK; b National Institute of Cardiology and Cardiovascular Surgery, Havana City, Cuba; c Institute of Hygiene, Epidemiology and Microbiology, Ministry of Public Health, Havana City, Cuba; d Cuban Commission against Smoking, Ministry of Public Health, Havana City, Cuba; e Directorate of Medical Records and Health Statistics, Ministry of Public Health, Havana City, Cuba; f Provincial Center of Hygiene, Epidemiology and Microbiology, Matanzas, Cuba; g Municipal Center of Hygiene, Epidemiology and Microbiology, Jagüey Grande, Matanzas, Cuba; h Municipal Center of Hygiene, Epidemiology and Microbiology, Colón, Matanzas, Cuba; i Municipal Center of Hygiene, Epidemiology and Microbiology, Camagüey, Cuba; j Medical Research Council Population Health Research Unit, Nuffield Department of Population Health, University of Oxford, Oxford, UK

## Abstract

**Background:**

The average age at which people start smoking has been decreasing in many countries, but insufficient evidence exists on the adult hazards of having started smoking in childhood and, especially, in early childhood. We aimed to investigate the association between smoking habits (focusing on the age when smokers started) and cause-specific premature mortality in a cohort of adults in Cuba.

**Methods:**

For this prospective study, adults were recruited from five provinces in Cuba. Participants were interviewed (data collected included socioeconomic status, medical history, alcohol consumption, and smoking habits) and had their height, weight, and blood pressure measured. Participants were followed up until Jan 1, 2017 for cause-specific mortality; a subset was resurveyed in 2006–08. We used Cox regression to calculate adjusted rate ratios (RRs) for mortality at ages 30–69 years, comparing never-smokers with current smokers by age they started smoking and number of cigarettes smoked per day and with ex-smokers by the age at which they had quit.

**Findings:**

Between Jan 1, 1996, and Nov 24, 2002, 146 556 adults were recruited into the study, of whom 118 840 participants aged 30–69 years at recruitment contributed to the main analyses. 27 264 (52%) of 52 524 men and 19 313 (29%) of 66 316 women were current smokers. Most participants reported smoking cigarettes; few smoked only cigars. About a third of current cigarette smokers had started before age 15 years. Compared with never-smokers, the all-cause mortality RR was highest in participants who had started smoking at ages 5–9 years (RR 2·51, 95% CI 2·21–2·85), followed by ages 10–14 years (1·83, 1·72–1·95), 15–19 years (1·56, 1·46–1·65), and ages 20 years or older (1·50, 1·39–1·62). Smoking accounted for a quarter of all premature deaths in this population, but quitting before about age 40 years avoided almost all of the excess mortality due to smoking.

**Interpretation:**

In this cohort of adults in Cuba, starting to smoke in childhood was common and quitting was not. Starting in childhood approximately doubled the rate of premature death (ie, before age 70 years). If this 2-fold mortality RR continues into old age, about half of participants who start smoking before age 15 years and do not stop will eventually die of complications from their habit. The greatest risks were found among adults who began smoking before age 10 years.

**Funding:**

UK Medical Research Council, Cancer Research UK, British Heart Foundation, US Centers for Disease Control and Prevention (CDC) Foundation (with support from Amgen).

## Introduction

According to the Global Adult Tobacco Surveys,^1^ at least 50 million adult smokers in low-income and middle-income countries started smoking before age 15 years, including about 6 million who started before age 10 years. Although substantial evidence exists that the younger individuals start smoking, the higher their subsequent risk of death, the effects of regularly smoking from childhood—and especially from early childhood—have not been well described. Direct evidence from large prospective studies, preferably in several different populations, is needed to quantify the effects of smoking regularly from early childhood and from later childhood.

Cuba has long been known for tobacco production, and about half of the men and a quarter of the women in the population smoke. Many of today's adult smokers started in childhood: according to the 2010 Cuban National Risk Factor Survey, 25% of smokers had started at ages 10–14 years, and another 4% had started before the age of 10 years.^2^ For comparison, analyses of the National Health Interview Survey^3^ data for 2018 indicate that 18% of daily adult smokers in the USA began smoking regularly at ages 10–14 years (an estimated 4·6 million people), and another 2% began before age 10 years (0·6 million).

As in most low-income or middle-income countries, previous attempts to quantify the mortality risks associated with smoking in Cuba have been limited to indirect methods, relying on risk estimates from other populations.^4^, ^5^ We report the association between smoking habits (focusing on the age when they started) and cause-specific premature adult mortality in a large prospective study of Cuban adults.

**Research in context**

**Evidence before this study**

We did a literature search to identify articles from prospective studies that reported on the association between smoking initiation in childhood (ie, before age 15 years) and mortality. We searched PubMed for articles published in any language between Jan 1, 1960, and Sept 8, 2019, using the terms “smoking” and “mortality” and “initiation” (or “onset” or “started”) and “prospective” (or “longitudinal” or “cohort). Although several studies compared the mortality effects of smoking initiation at younger ages with those of initiation at older ages, only a few small-scale studies directly assessed the effect of starting to smoke in childhood, and none of these reported the effects of smoking initiation from as young as age 10 years (or younger). Cross-sectional evidence suggests that starting to smoke before age 15 years is common in many populations worldwide, yet substantial uncertainty exists about the health effects of starting to smoke from such a young age.

**Added value of this study**

In this Cuban study of nearly 150 000 adults, a third of participants who smoked regularly at recruitment began smoking in childhood. Starting to smoke in childhood approximately doubled the risk of premature adult mortality, and starting in early childhood (ie, before age 10 years) was associated with an even greater excess risk (nearly 3 times the excess risk as starting at age 15 or older). Smoking cessation was uncommon in this population, but participants who quit smoking before the age of about 40 years avoided most of the excess risk associated with prolonged smoking. A re-examination of US data relating adult mortality to starting to smoke regularly before age 15 years revealed similar patterns.

**Implications of all the available evidence**

Our findings indicate that the many millions of adult smokers worldwide who began smoking regularly in childhood, and particularly in early childhood, will be at especially high risk of premature mortality if they continue to smoke through adult life. However, even among smokers who started very young, the sooner they quit, the lower their risk of premature death; and those who quit successfully before age 40 years (and preferably well before that) avoided most of the excess risk of premature death that would otherwise be caused by prolonged smoking.

## Methods

### Study design and participants

Cuban mortality patterns and the characteristics of the participants in this study have been described elsewhere.^6^ Briefly, men and women aged 30 years or older were recruited into a prospective cohort study from five provinces in Cuba.^6^ Within each of these provinces, family medical clinics were randomly selected (215 medical clinics were approached, and none refused to participate) and clinic staff (mostly physicians) sought to recruit all adults living in the clinic's catchment area (74% of adults agreed to participate), recording age, sex, education, occupation, medical history, self-reported alcohol consumption, and smoking history. Ever-smokers were asked at what age they had first smoked regularly (ie, on most days); the average number of cigarettes and cigars smoked per day in the past month; and, among those who had not smoked in the past month, the age at which they had last stopped (the questionnaire is available in the appendix p 22). Participants were then invited to their local clinic for measurement of height, weight, and blood pressure. Participants aged 70 years or older at recruitment were excluded from the main analyses, as were the few with incomplete data on smoking or covariates. To assess the usual mean cigarette consumption during the follow-up period in each separate category of baseline-reported habits, participants in some areas were resurveyed in 2006–08 (on average 6 years after recruitment) with the same procedures as at recruitment. The study has been approved by the National Institute of Cardiology Ethics Committee (Cuba).

### Mortality follow-up

Participants were followed up to Jan 1, 2017. Follow-up was censored at the date of death, the end of the risk period under consideration, date of loss to follow-up, or at the end of the follow-up period. Deaths were identified annually through linkage^6^ to the Cuban Public Health Ministry's national mortality records by use of participants’ national identification numbers, names, and birth dates. In Cuba, almost all deaths are certified by a doctor, with the underlying and contributing causes of death coded according to standard WHO recommendations. Coders used the ninth edition of the International Classification of Diseases (ICD-9) for deaths between 1996 and 2000 and the tenth edition (ICD-10) for deaths between 2001 and 2017 (appendix p 15). Although participants who emigrated were lost to follow-up, emigration from Cuba is very low (<1% per annum).^6^

### Statistical analysis

We used Cox regression to calculate mortality rate ratios (RRs) comparing smokers in various categories of smoking at recruitment with never-smokers at recruitment. The main analyses were of RRs for premature mortality (ie, before age 70 years), adjusted for age (in 5-year groups of age at risk, 30–69 years), sex, education completed (four groups: did not complete primary education, primary education, secondary education, and high school or university), province (five provinces), alcohol consumption (none, once per week, once or more per week), and body-mass index (BMI; in six groups with cutoff points at 20, 22·5, 25, 27·5, and 30 kg/m^2^). To examine whether the RRs varied with time, the adjusted RRs were estimated separately for each 5-year period of follow-up.

RRs compared various categories of current smokers or ex-smokers with never-smokers. In current cigarette smokers, we divided participants by the age at which they started smoking into categories of 5–9 years, 10–14 years, 15–19 years, and 20 years or older; and by the amount they smoked at the baseline survey in categories of less than ten, 10–19, 20, or more than 20 cigarettes per day (ignoring the small amount of cigar smoking among cigarette smokers, because only a small proportion of cigarette smokers smoked cigars, and those who did smoked few cigars on average). We chose these categories partly to accommodate strong digit preference (the tendency for participants to choose round numbers such as 10 or 20; appendix pp 5–7). Age when stopping smoking was categorised as 25–34 years, 35–44 years, and 45–54 years. For analyses by age when stopping smoking, we excluded participants who stopped smoking at ages older than 54 years to reduce the effect of quitting because of illness and excluded those who quit before they were 25 years old because there were too few participants in this category for reliable analyses.

Participants with chronic disease at recruitment (including ischaemic heart disease, stroke, cirrhosis, cancer, chronic kidney disease, or chronic obstructive pulmonary disease [COPD]) were included in the analyses of current smokers versus never-smokers, but were excluded from the analyses of ex-smokers versus current or never-smokers (to avoid reverse causality, whereby previous disease might have led to quitting). Participants who quit during the 5 years before recruitment were also excluded from analyses assessing the effects of stopping smoking, partly to help ensure that the analyses would assess the effects of long-term cessation, and partly because recent ex-smokers might have been more susceptible to a relapse (especially since, at the time of the baseline survey, the economy of Cuba was not long past the hardships of the Special Period in the 1990s). To validate these findings in an external population, we examined the relevance of age when starting to smoke to all-cause mortality in the USA using the National Health Interview Surveys (1997–2014) linked to the National Death Index (details on background and methods in the appendix, pp 24–25).

For each category of current smoking at recruitment, we plotted RRs by amount smoked against the mean number of cigarettes smoked per day at resurvey among participants who continued to smoke. This was done to relate the RRs in each of the baseline-defined smoking categories to an estimate of the so-called usual (ie, long-term average) number of cigarettes smoked per day during follow-up in those categories.

We used the variances and covariances of the log RRs in each category (except the reference group, with RR 1) to estimate the variance of the log risk in each group (including the reference group). We then used this group-specific variance to calculate the group-specific 95% CI, which reflects the amount of data in that single group or category alone; this method allows pairwise comparisons between any two groups (as opposed to only pairwise comparisons with the reference group).^7^ When plotting graphs, the group-specific CIs are presented, but when comparing two categories directly (eg, current versus never smoking), conventional CIs are used.

The fraction of deaths attributable to smoking (ie, the population-attributable fraction) was estimated as P–P/RR, where P is the prevalence of smoking among participants dying of a given disease and RR is the disease-specific RR in ever-regular (includes those who currently smoked at recruitment and those who reported having formerly smoked regularly but had quit by recruitment) versus never-smokers.^8^ All analyses were done with SAS, version 9.4, or R, version 3.1.1.

### Role of the funding source

The funders of the study had no role in study design, data collection, data analysis, data interpretation, or writing of the report. BT, NAR, BL, RP, SL, and ADH had full access to the data and analyses and shared final responsibility for the decision to submit for publication. The corresponding author had full access to all the data in the study and had final responsibility for the decision to submit for publication.

## Results

Between Jan 1, 1996, and Nov 24, 2002, 146 556 adults were recruited into the study. Of these, 24 159 were excluded from the main analyses because they were aged 70 years or older at recruitment. An additional 3557 were excluded because of missing information on smoking or covariates, leaving 118 840 (52 524 men and 66 316 women) in the main analyses (appendix p 2). 3578 participants included in the main analyses provided smoking information at resurvey. Mean age at recruitment was 50 years (SD 10), and 25 140 (48%) of 52 524 men and 25 626 (39%) of 66 316 women had completed high school (table). At recruitment, 27 264 (52%) men and 19 313 (29%) women reported current smoking (with 44 042 [95%] smoking some cigarettes), and 16 360 (31%) men but only 3228 (5%) women reported drinking alcohol in most weeks. Mean systolic blood pressure was 124 mm Hg (SD 15), and mean BMI was 24 kg/m^2^ (SD 4).

**Table:** Characteristics of the 118 840 participants included in the mortality analyses, by smoking pattern and age when starting to smoke

		**Never-smokers**	**Ex-smokers**	**Smokers of cigars only**	**Smokers of cigarettes by age they started smoking (years)**					**Total**
					Any age	5–9	10–14	15–19	≥20	
Number of participants		65 186	7077	2535	44 042	1724	13 432	19 165	9721	118 840
Age, years		51 (10)	53 (10)	57 (9)	49 (9)	52 (10)	50 (10)	48 (9)	49 (9)	50 (10)
Sex										
	Men	21 515 (33%)	3745 (53%)	2184 (86%)	25 080 (57%)	1129 (65%)	8210 (61%)	11 323 (59%)	4418 (45%)	52 524 (44%)
	Women	43 671 (67%)	3332 (47%)	351 (14%)	18 962 (43%)	595 (35%)	5222 (39%)	7842 (41%)	5303 (55%)	66 316 (56%)
Completed high school		28 483 (44%)	3007 (42%)	629 (25%)	18 647 (42%)	378 (22%)	4664 (35%)	8850 (46%)	4755 (49%)	50 766 (43%)
Consume alcohol weekly		5611 (9%)	1201 (17%)	670 (26%)	12 106 (27%)	478 (28%)	3909 (29%)	5559 (29%)	2160 (22%)	19 588 (16%)
BMI, kg/m^2^		25 (4)	25 (4)	25 (4)	24 (4)	24 (4)	24 (4)	24 (4)	24 (4)	24 (4)
Systolic blood pressure, mm Hg		124 (15)	126 (16)	128 (16)	123 (15)	124 (17)	123 (15)	123 (14)	124 (15)	124 (15)
Baseline smoking habits										
	Age when starting to smoke (years)	NA	17 (6)	17 (7)	17 (5)	8 (1)	12 (1)	17 (1)	24 (6)	17 (5)
	Number of cigarettes per day*	NA	NA	NA	15 (9)	18 (11)	17 (9)	15 (8)	13 (8)	12 (10)†
	Cigar smokers*	NA	NA	2535 (100%)	3144 (7%)	205 (12%)	1171 (9%)	1247 (7%)	521 (5%)	5679 (5%)
Resurvey smoking habits (n=3578)‡										
	Current smoker at resurvey	100 (4%)	24 (26%)	60 (80%)	984 (83%)	74 (86%)	280 (83%)	411 (86%)	219 (80%)	1168 (33%)
	Number of cigarettes per day among those still smoking	NA	NA	NA	17 (11)*	19 (10)	18 (10)	16 (9)	13 (9)	16 (10)
	Number of cigarettes per day among all resurveyed	0 (3)	4 (9)	3 (7)	13 (11)	16 (12)	14 (11)	13 (10)	10 (10)	5 (9)

Data are n (%) or mean (SD). Participants aged 70 years or older at recruitment and those with missing information on smoking or covariates were excluded. BMI=body-mass index.

* Smokers of cigars consumed only 3·2 (SD 3·1) cigars per day, and the few cigarette smokers who also smoked cigars consumed 3·8 (SD 4·5) cigars per day.

† Mean (SD) number of cigarettes among all participants.

‡ Results from the 3578 participants who were resurveyed (at about 6 years after the baseline survey); of these participants at baseline, 2233 had never smoked, 91 had been ex-smokers, 75 had smoked only cigars, and 1179 had smoked cigarettes (mean 16 cigarettes per day).

**Figure 1:**
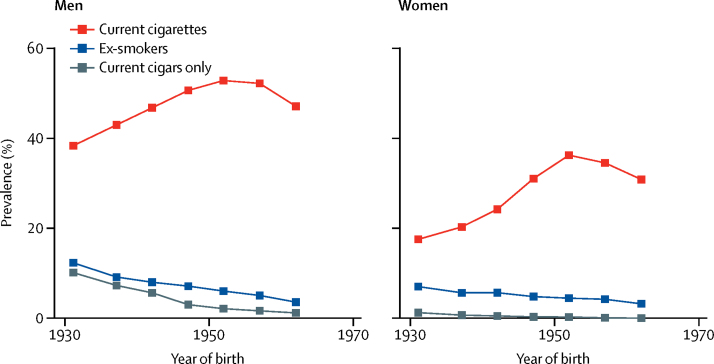
Smoking prevalence in the baseline survey (1996–2002) by year of birth Proportion of participants at baseline who were current smokers of cigarettes, current smokers of cigars only, or ex-smokers (of either cigarettes or cigars). Years of birth are divided into the following categories: before 1935, 1935–39, 1940–44, 1945–49, 1950–54, 1955–59, and 1960 or after.

For both men and women, smoking prevalence at baseline was highest among those born in the 1950s and was slightly lower among those born in the 1960s (figure 1). Stopping smoking was uncommon in both sexes: among those who reported having smoked regularly, only 3745 (12%) of 31 009 men and 3332 (15%) of 22 645 women had quit at baseline. Most current smokers reported smoking cigarettes (3144 [7%] of 44 042 in combination with cigars; figure 1), but a few (mostly older) men and even fewer women reported only smoking cigars (appendix pp 16–17). Current smokers consumed a mean of 15 cigarettes per day (16 cigarettes [SD 9] in men and 13 cigarettes [SD 8] in women) and had started to smoke at a mean age of 17 years (16 years [SD 4] in men and 17 years [SD 6] in women; appendix pp 3–4, 6). About a third of current cigarette smokers reported having started before age 15 years: 1724 (4%) of 44 042 at ages 5–9 years and 13 432 (30%) at ages 10–14 years.

In comparison with never-smokers, current smokers were 3 times as likely to drink alcohol in most weeks and had similar blood pressure, but slightly lower BMI. Participants who had started smoking before age 15 years were, on average, older and had completed substantially less education than those who had started later (table). At the partial resurvey, about 6 years after the baseline survey, about a sixth of participants who had been smokers at baseline reported having stopped. The mean daily consumption of cigarettes among all who were still smoking at resurvey had changed little since the baseline survey, but regression towards this overall mean was observed; hence, among participants who, at baseline, had reported being lighter smokers, the average consumption reported at resurvey had increased, whereas among those who had reported being heavier smokers, consumption had decreased (appendix p 18). For each category of cigarette consumption at baseline, the mean consumption at resurvey was taken as the usual (ie, long-term average) cigarette consumption.

Among the 118 840 adults contributing to the main analyses, there were 1·7 million person-years of follow-up at ages 30–69 years (mean 14 years per person, SD 6), during which 8571 deaths occurred: 3072 due to vascular causes (1607 ischaemic heart disease and 1465 stroke or other vascular cause), 2950 due to neoplastic causes (1064 lung or upper aero-digestive cancer and 1886 other type of cancer), 656 due to respiratory causes (314 COPD and 342 other respiratory cause), 1395 due to other medical causes, and 498 due to external causes.

**Figure 2:**
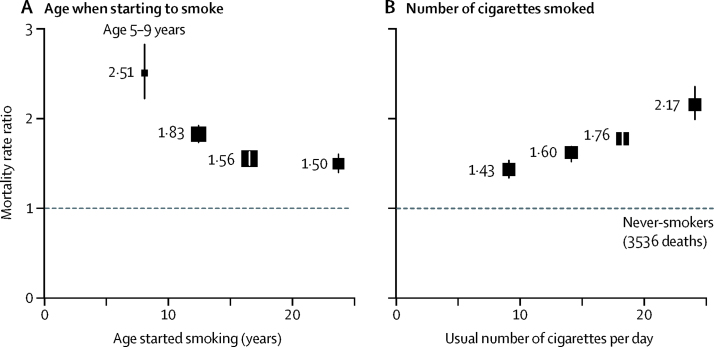
All-cause mortality at ages 30–69 years for cigarette smokers versus never-smokers by age when starting to smoke (A) and by usual number of cigarettes smoked per day (B) Box area is inversely proportional to the variance of the log risk. Error bars denote group-specific 95% CIs. Analyses were adjusted for age, sex, education, alcohol consumption, body–mass index, and province. (A) Age at which participants started smoking regularly was divided into the following categories: 5–9 years, 10–14 years, 15–19 years, and 20 years or older. (B) Participants were categorised by the number of cigarettes smoked per day at baseline (<10, 10–19, 20, and >20) and the mortality rate ratios plotted against the mean number of cigarettes smoked per day at resurvey in these baseline-defined groups.

**Figure 3:**
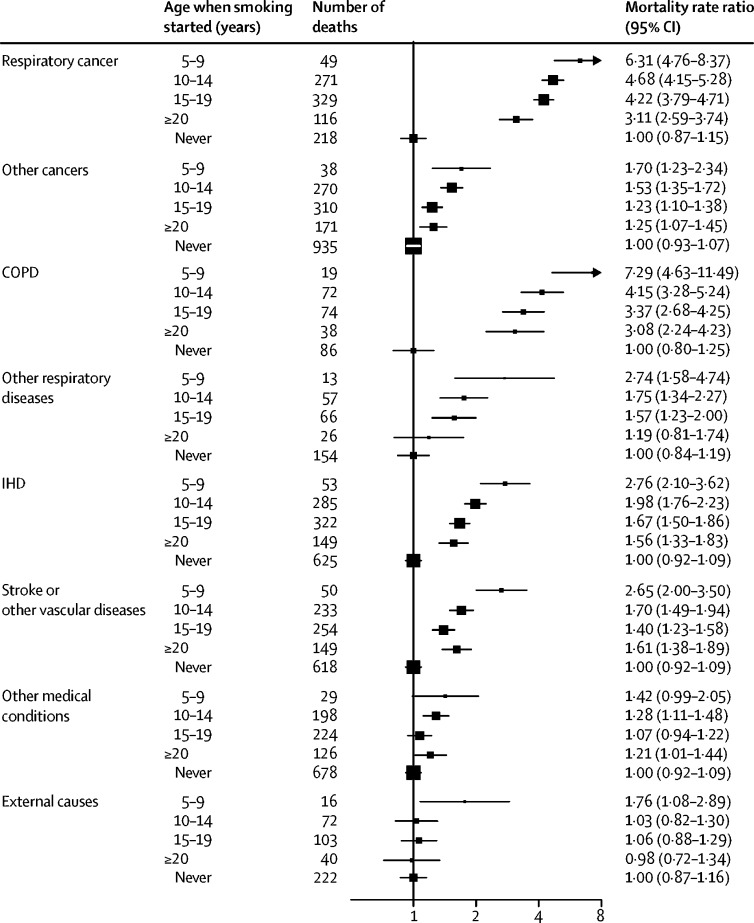
Cause-specific mortality at ages 30–69 years for cigarette smokers versus never-smokers by age when starting to smoke Box area is inversely proportional to the variance of the log risk. Error bars denote group-specific 95% CIs. Analyses were adjusted for age, sex, education, alcohol consumption, body-mass index, and province. COPD=chronic obstructive pulmonary disease. IHD=ischaemic heart disease.

Compared with never-smokers, the all-cause mortality RR at ages 30–69 years for current cigarette smokers was 1·66 (95% CI 1·58–1·74) and was similar in both sexes. However, this RR depended strongly on the age at which smoking had started and on the number of cigarettes smoked (Figure 2, Figure 3; appendix p 8). All-cause mortality RR was highest in participants who had started smoking at ages 5–9 years (RR 2·51, 95% CI 2·21–2·85), followed by ages 10–14 years (1·83, 1·72–1·95), 15–19 years (1·56, 1·46–1·65), and ages 20 years or older (1·50, 1·39–1·62). Similarly, all-cause mortality RR was lowest for participants who had reported smoking fewer than ten cigarettes per day at baseline (RR 1·43, 95% CI 1·33–1·55), followed by ten to 19 cigarettes (1·60, 1·50–1·71), 20 cigarettes (1·76, 1·65–1·87), and more than 20 cigarettes (2·17, 1·97–2·38). These relationships were similar when men and women were considered separately (appendix pp 9–10). The associations were little changed by the following: controlling age when smoking started and the amount smoked per day for each other; by excluding participants with chronic disease at baseline; or by excluding current smokers who were known from the baseline survey to have quit smoking previously for at least 1 year and then restarted (appendix pp 11–12). Furthermore, the comparison of RRs over the follow-up period provided no evidence that the proportional hazards assumption had been violated.

For several causes of death, we observed dose-response relationships with age when starting to smoke and with the number of cigarettes smoked per day. These causes included respiratory cancer (lung and upper aero-digestive), ischaemic heart disease, and COPD (figure 3, appendix p 8). Although exclusive cigar smoking was uncommon at the baseline survey, it was associated with a significant increase in all-cause mortality (RR 1·27, 1·11–1·47; appendix p 13), but this increase was only half as great as that associated with cigarette smoking.

**Figure 4:**
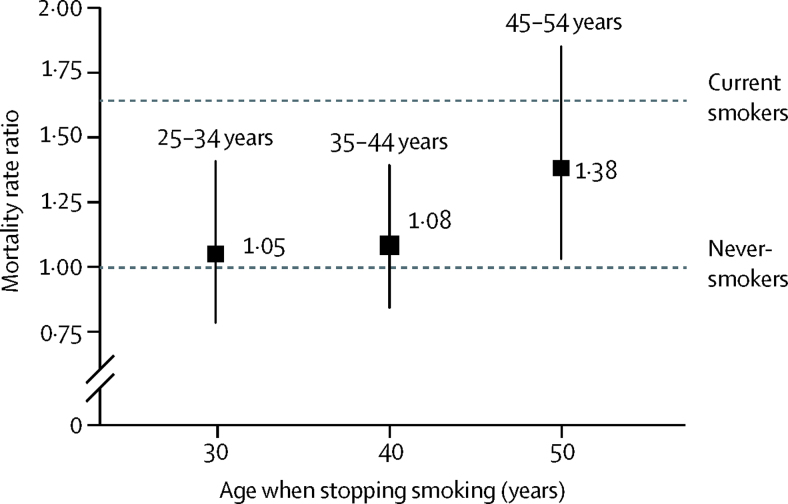
All-cause mortality at ages 30–69 years for ex-cigarette smokers versus never-smokers by age when stopping smoking Box area is inversely proportional to the variance of the log risk. Error bars denote group-specific 95% CIs. Analyses adjusted for age, sex, education, alcohol consumption, body–mass index, and province. Age when stopping smoking was divided into the following groups: 25–34 years (45 deaths), 35–44 years (61 deaths), and 45–54 years (45 deaths).

Ex-smokers who had reported at the baseline survey that they had quit at about age 40 years (ie, between ages 35 and 44 years) appeared to have little of the excess mortality before age 70 years of participants who were still current smokers at recruitment (figure 4). In people without chronic disease at baseline, the all-cause RRs comparing those who had stopped smoking with never-smokers were RR 1·05 (95% CI 0·78–1·41) for those who quit at ages 25–34 years, 1·08 (0·84–1·39) at ages 35–44 years, and 1·38 (1·03–1·86) at ages 45–54 years, compared with 1·64 (1·56–1·73) for current smokers at baseline (some of whom will have quit during follow-up). Insufficient deaths occurred for us to assess reliably the effects of quitting on specific diseases or the joint effects on mortality of age when starting to smoke with age when stopping smoking.

**Figure 5:**
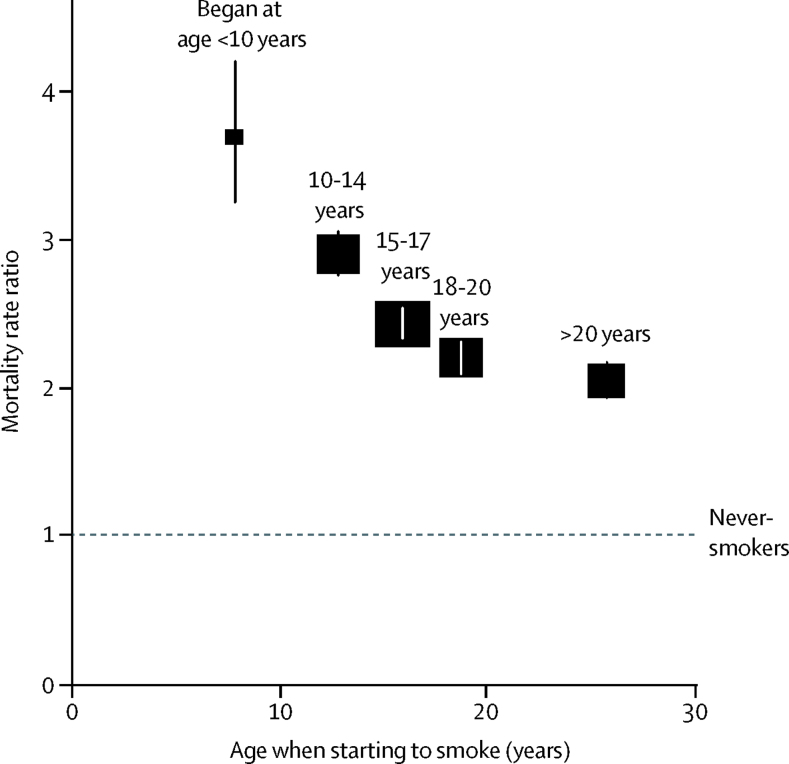
All-cause mortality in the USA at ages 30–69 years for cigarette smokers versus never-smokers by age when starting to smoke Box area is inversely proportional to the variance of the log risk. Error bars denote group-specific 95% CIs. Age when starting to smoke regularly was divided into the following categories: younger than 10 years (233 deaths), 10–14 years (1566 deaths), 15–17 years (2237 deaths), 18–20 years (1539 deaths), and older than 20 years (1116 deaths). Data sources were the US National Health Interview Surveys and US National Death Index. Analyses were adjusted for age at risk, sex, race, education, alcohol consumption, and region.

Among 326 456 adults aged 30–69 years included in analyses of the US National Health Interview Survey prospective study, there were 2·2 million person-years of follow-up and 13 389 deaths (6698 among never-smokers and 6691 among daily smokers). The fully-adjusted all-cause mortality RR comparing daily versus never-smokers was 3·70 (95% CI 3·25–4·21) for those who had started before age 10 years, 2·91 (2·76–3·06) for those who had started at ages 10–14 years, 2·43 (2·33–2·54) for those who had started at ages 15–17 years, 2·20 (2·09–2·32) for those who had started at ages 18–20 years, and 2·04 (1·93–2·17) for those who had started after age 20 years (figure 5; further results and discussion in the appendix, pp 21, 24–25).

## Discussion

In this large-scale prospective study in Cuba, many participants had started to smoke in childhood, either at ages 10–14 years or even before age 10 years. Starting in childhood (before age 15 years) approximately doubled the risk of premature adult mortality, and starting in early childhood (before age 10 years) was associated with even greater excess risk (nearly 3 times the excess risk as that of starting at age 15 or older). Stopping smoking was uncommon in this population, but those who quit smoking before the age of about 40 years avoided most of the excess risk associated with prolonged smoking.

Given the diseases we observed to be associated with smoking in Cuba, together with the evidence from similar studies in North America and Europe, it is reasonable to assume that the excess mortality among smokers was largely caused by the adverse effects of smoking. As such, smoking was likely to be the cause of about half of all premature adult deaths among participants who began smoking in childhood. Even among smokers who had begun after age 20 years, smoking was a probable cause of about a third of all premature adult deaths.

The excess mortality caused by smoking regularly since early adulthood is substantial, but the excess mortality caused by smoking since childhood is even greater. In this cohort, the mean age when participants started to smoke was 17 years, but more than a third of the smokers had begun before age 15 years. By contrast with some other middle-income countries, where the age of starting to smoke has decreased in more recent birth cohorts,^9^, ^10^ the proportion of smokers in Cuba who had begun smoking in childhood (or in early childhood) was broadly similar for those born in the 1940s, 1950s, and 1960s. The proportion of smokers who began before age 15 years has been documented in several countries,^5^, ^11^, ^12^ including Cuba^2^ and the USA.^13^ The large excess mortality caused by starting to smoke in early adulthood is well recognised,^14^ but the effects of starting before age 15 years (and particularly before age 10 years) have not been adequately investigated. Therefore, our findings from Cuba led us to re-examine US data relating adult mortality to participants starting to smoke regularly before age 15 years, which revealed similar effects of starting to smoke at younger than at older ages.

Most smokers in Cuba smoke cigarettes,^2^, ^15^ and those who smoked cigarettes had higher risk of premature death than the few who smoked only cigars. Although exclusive cigar smokers had higher risks of premature death than those of never-smokers, we were not able to assess how many of them had at other times smoked appreciable numbers of cigarettes. The higher risk associated with smoking cigarettes rather than cigars is consistent with previous studies in high-income countries.^16^, ^17^

Smoking was more prevalent among men than among women but, for some diseases, the smoker versus never-smoker mortality RR was greater in women than in men. Both in men and in women, smoking accounted for about half of all deaths from respiratory cancer (lung or upper aero-digestive tract) and a quarter of all deaths from ischaemic heart disease (appendix p 19). Overall, about a quarter (27%) of all premature adult deaths in men and a fifth (19%) of premature adult deaths in women were attributable to smoking.

In this study of Cuban adults, lung cancer mortality rates recorded among men and women who had never smoked were about 3 times as great as those in US prospective studies. The reason for this is unclear but is perhaps because some deaths with pulmonary metastases from another site were miscertified as deaths from primary lung cancer, which would attenuate the smoker versus never-smoker mortality ratio (appendix p 20). For other major smoking-related causes (ischaemic heart disease, COPD, and stroke), the smoker versus never-smoker mortality RR were similar to those reported from several other middle-income countries and somewhat lower than those in contemporary US studies, but they are similar to those seen in a large study of US mortality during the 1960s.^10^, ^13^, ^18^

Smoking is common in Cuba but quitting is not, despite various major economic crises over the past few decades (appendix p 14).^19^ Although Cuba has a strong national focus on primary care and preventive medicine, which has led to substantial progress on hypertension control,^20^ similar progress has not yet been achieved on tobacco control. Only Cuba, the USA, and a handful of other countries have not yet ratified the Framework Convention on Tobacco Control, which has spurred progress on tobacco control in many other populations. The relatively high smoking prevalence and low rate of cessation in this population highlight an area of considerable opportunity to improve public health through prevention.

However, among participants who quit smoking, the health benefits were substantial. Quitting smoking by about age 40 years avoided most of the excess risk of premature death associated with continued smoking. Although the precision of these estimates is constrained by the low number of ex-smokers in this study, the benefits of quitting were similar to those reported in studies elsewhere with much larger numbers of ex-smokers.^14^, ^21^ Furthermore, these apparent benefits might underestimate the true benefits of quitting permanently, because some who had quit might have done so because of ill health (as opposed to quitting by choice while still healthy), and some of those who had quit before the baseline survey had restarted by the time of the resurvey.

A key strength of this study is the large sample size, which allowed assessment of the hazards of smoking and benefits of quitting in a population in which childhood initiation of smoking was common. Although smoking information relied on a single report at the initial survey, a partial resurvey about 6 years later helped to assess the usual smoking habits in each baseline-defined smoking category. The high participation rate in the study, which sampled adults from multiple provinces across the country, should limit the potential effect of selection bias. One noteworthy limitation of our study is that the baseline study took place during the Special Period in Cuba, when considerable economic hardship was prevalent and might have temporarily altered smoking patterns. Another limitation is that, although sensitivity analyses assessed the effect of excluding all who reported previous chronic disease at baseline, reverse causality might still have led to some underestimation of the hazards of smoking or benefits of quitting. Additionally, some misclassification in the age at which participants started smoking and in the amount smoked per day was possible, which might have attenuated the differences in relative risks between categories. Furthermore, some residual confounding cannot be ruled out.

Starting to smoke regularly in adulthood substantially increased the risk of premature death in later decades, but starting in childhood (before age 15 years) approximately doubled this excess risk, and starting in early childhood (before age 10 years) approximately tripled it. Among adult smokers, this study reinforces the findings from other populations that the sooner smokers quit, the lower their risk of premature death, and that those who quit successfully before age 40 years (and preferably well before that) avoid most of the excess risk of premature death that would otherwise be caused by smoking.
