# Supplementary material for: Association of childhood smoking and adult mortality: prospective study of 120 000 Cuban adults
Source: Lancet Glob Health. 2020 May 21;8(6):e850–7. doi: 10.1016/S2214-109X(20)30221-7 (PMC7248573; doi:10.1016/S2214-109X(20)30221-7)
Supplement: Supplementary appendix [file mmc1.pdf]

THE LANCET
Global Health

Supplementary appendix

This appendix formed part of the original submission and has been peer reviewed. We post it as supplied by the authors.

Supplement to: Thomson B, Armas Rojas N, Lacey B, et al. Association of childhood smoking and adult mortality: prospective study of 120 000 Cuban adults. *Lancet Glob Health* 2020; **8:** e850–59.

Supplementary Appendix
Association of childhood smoking and adult mortality: prospective study of 120 000 Cuban adults

Thomson B, Armas Rojas N, Lacey B et al.

https://www.ctsu.ox.ac.uk/research/the-cuba-prospective-study

SUPPLEMENTARY FIGURES **Page**

**Figure 1:**	Flow chart of participant exclusions	**2**
**Figure 2:**	Mean consumption and age started smoking, by year of birth	**3**
**Figure 3:**	Prevalence of smoking amount (number of cigarettes/day), by sex and year of birth	**4**
**Figure 4:**	Distribution of reported number of cigarettes smoked per day per current smoker at baseline	**5**
**Figure 5:**	Prevalence of age started smoking, by sex and year of birth	**6**
**Figure 6:**	Distribution of reported age started smoking among current smokers at baseline	**7**
**Figure 7:**	Cause-specific mortality at ages 30-69 years, cigarette smoker vs never-smoker, by amount smoked at baseline	**8**
**Figure 8:**	All-cause mortality at ages 30-69 years, by sex and age started smoking	**9**
**Figure 9:**	All-cause mortality at ages 30-69 years, by sex and amount smoked	**10**
**Figure 10:**	All-cause mortality at ages 30-69 years, by age started smoking and amount smoked, after mutual adjustment	**11**
**Figure 11:**	All-cause mortality at ages 30-69 years, by age started smoking and amount smoked. Effect of excluding those who had quit for one or more years	**12**
**Figure 12:**	All-cause mortality at ages 30-69 years for cigar only and cigarette smokers versus never-smokers	**13**
**Figure 13:**	Mean number of cigarettes sold per adult per day in Cuba, 1980-2015	**14**

SUPPLEMENTARY TABLES

**Table 1:**	ICD-9 and ICD-10 codes for mortality endpoints	**15**
**Table 2:**	Characteristics of the 52 524 male participants included in the mortality analyses, by smoking pattern and age started smoking	**16**
**Table 3:**	Characteristics of the 66 316 female participants included in the mortality analyses, by smoking pattern and age started smoking	**17**
**Table 4:**	Characteristics at resurvey about 6 years later, by baseline smoking status and amount smoked	**18**
**Table 5:**	Cause-specific population-attributable fractions (PAF) at ages 30-69 years, by sex	**19**
**Table 6:**	Comparisons between the never-smoker lung cancer mortality rates in the Cuba Prospective Study during 1996-2016 and the American Cancer Society prospective study during 1984-88	**20**
**Table 7:**	Baseline characteristics of 1997-2014 US National Health Interview Surveys	**21**

QUESTIONNAIRES

Spanish original	**22**
English translation	**23**

SUPPLEMENTARY ANALYSES

Brief report on analyses of the 1997-2014 US National Health Interview Surveys	**24-25**

Address for correspondence: cuba.prospective.study@ndph.ox.ac.uk

Supplementary Figure 1: Flow chart of participant exclusions

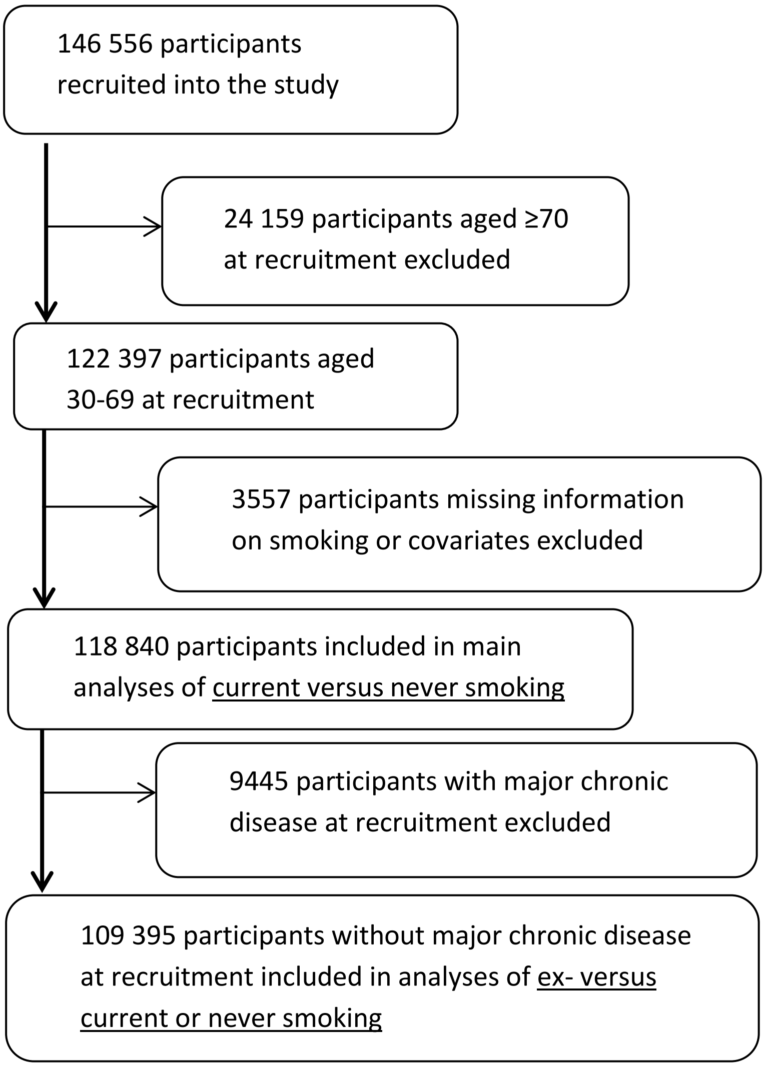

Supplementary Figure 2:
Mean consumption and age started smoking, by year of birth

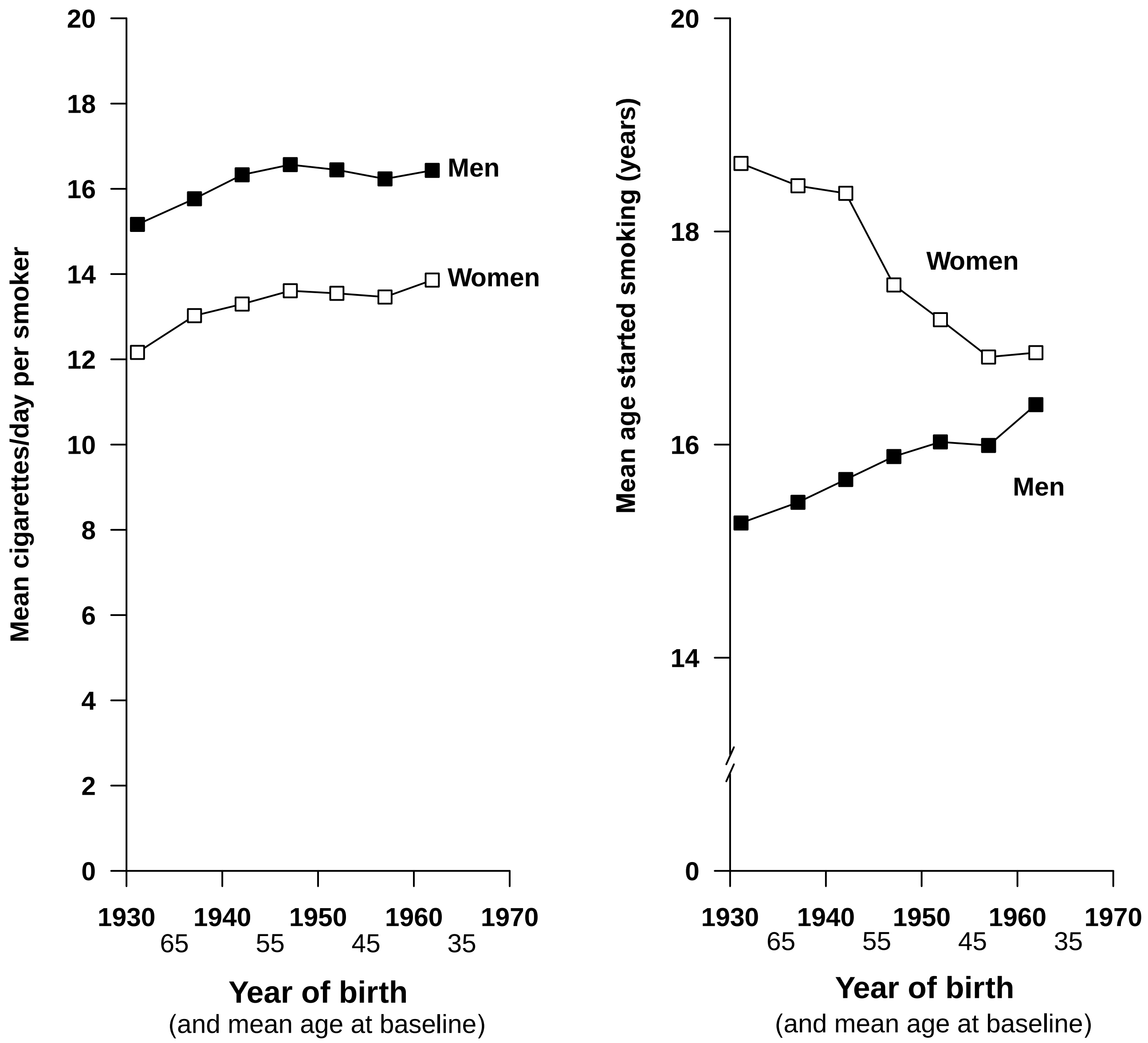

Supplementary Figure 3:
Prevalence of smoking amount (number of cigarettes/day), by sex and year of birth

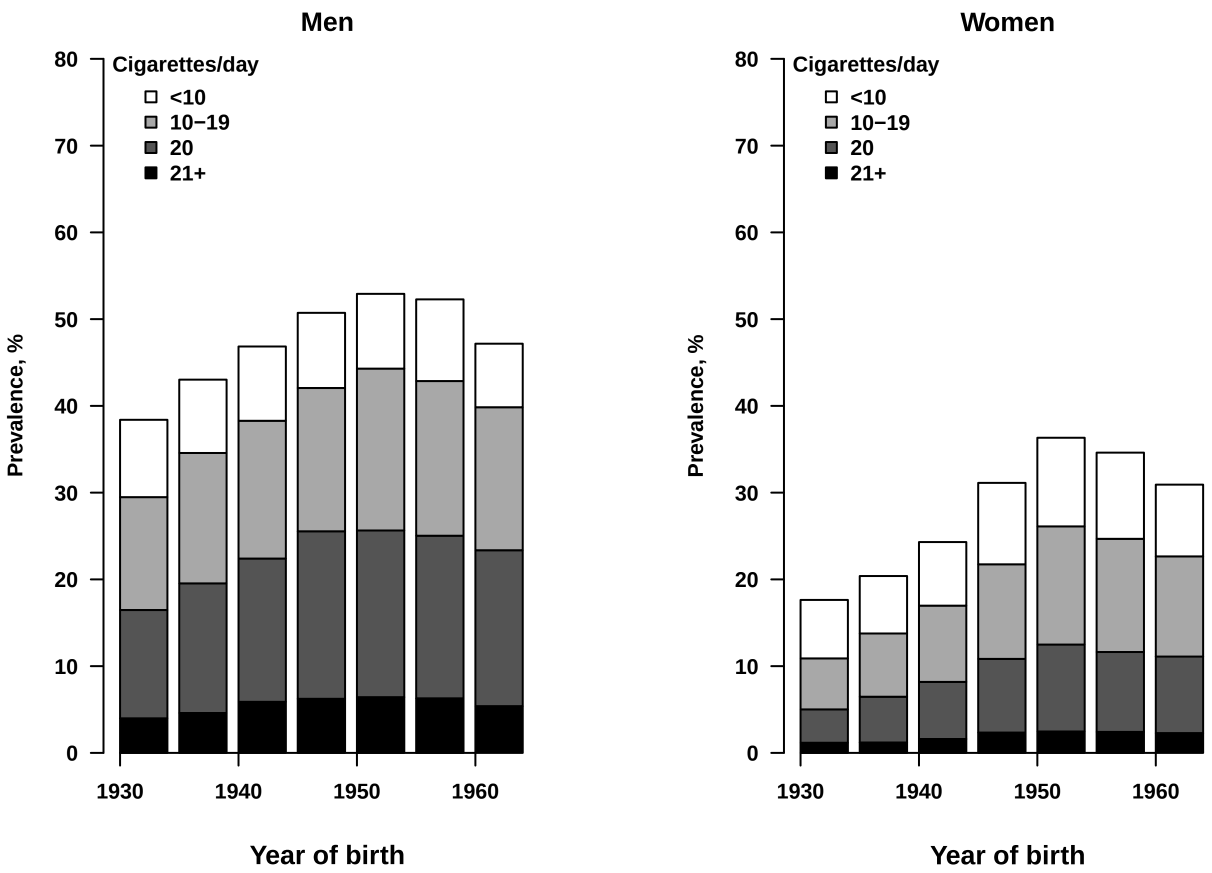

Supplementary Figure 4:
Distribution of reported number of cigarettes smoked per day per current smoker at baseline

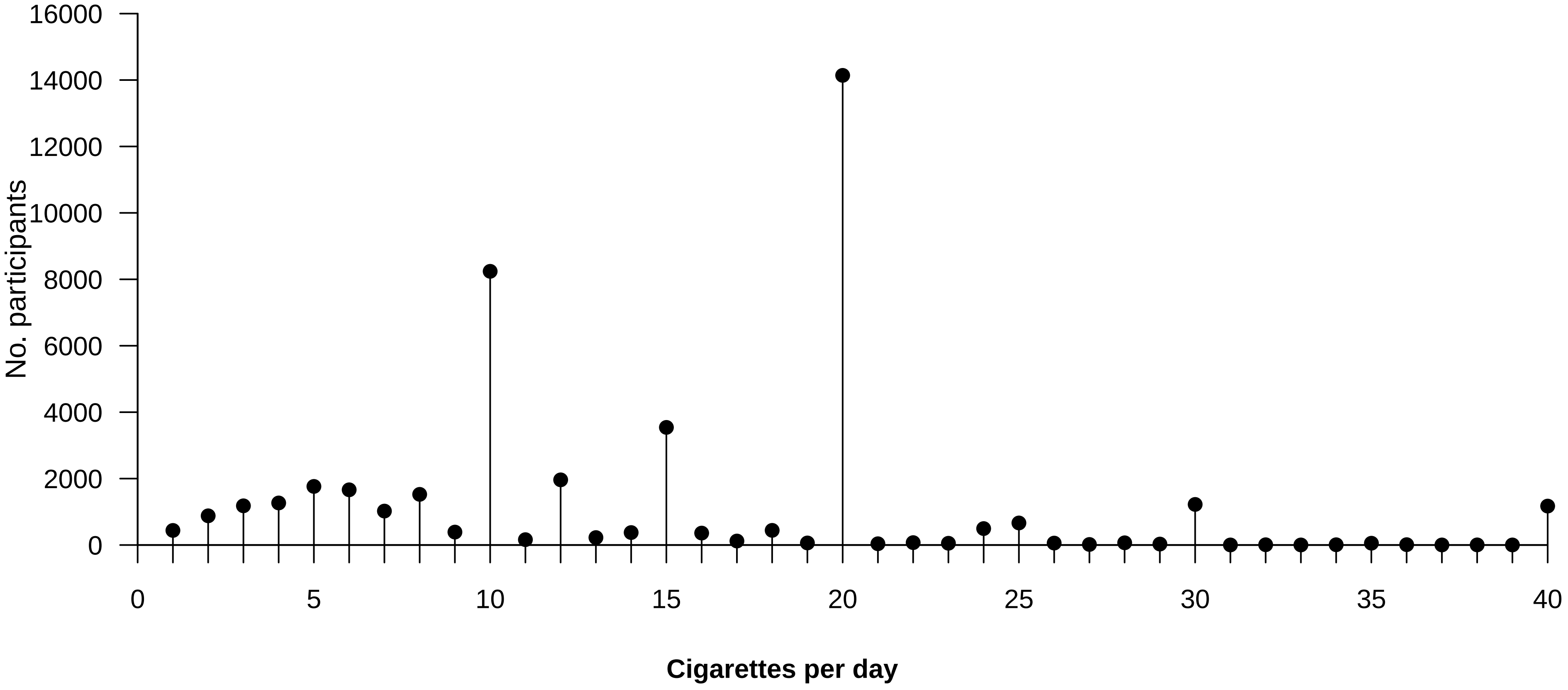

Note: the <1% of smokers who reported smoking >40 cigarettes/day not pictured.

Supplementary Figure 5:
Prevalence of age started smoking, by sex and year of birth

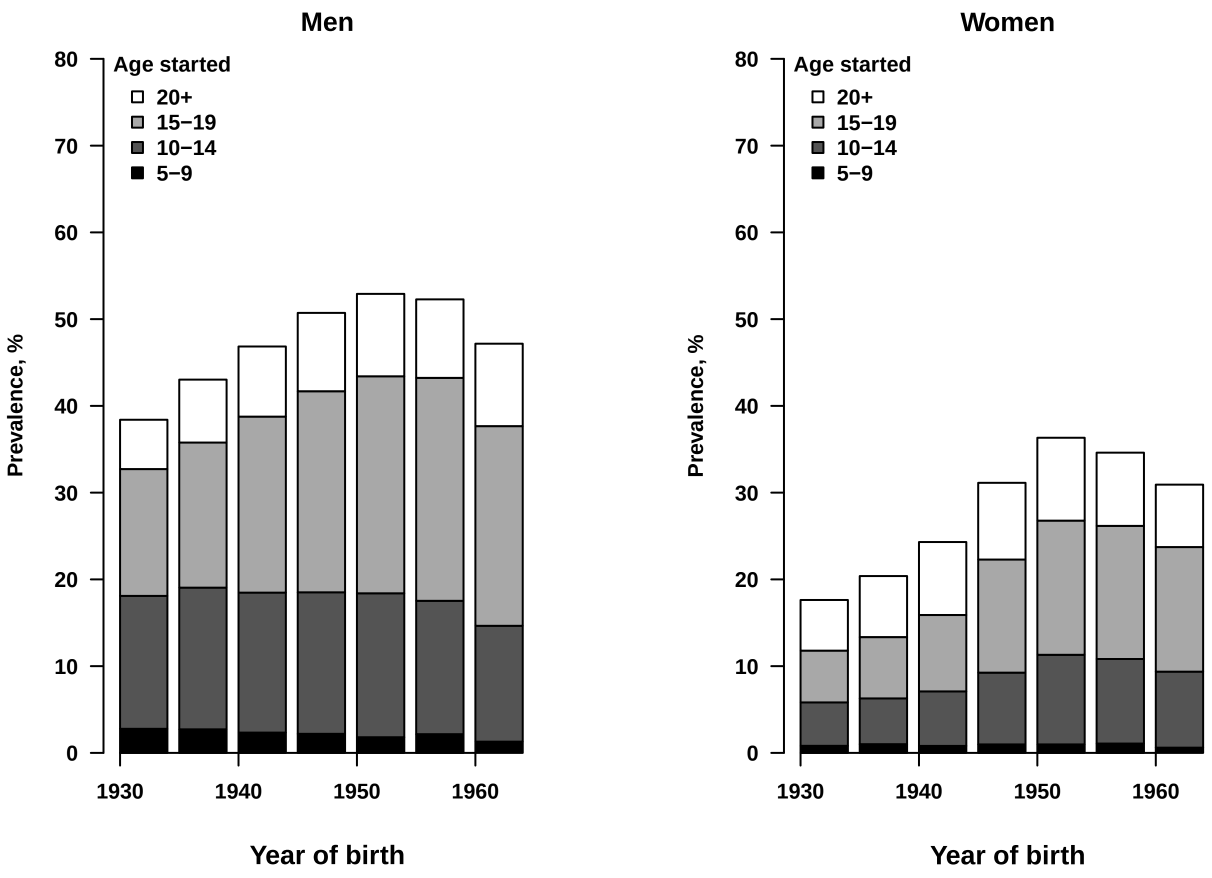

Supplementary Figure 6:
Distribution of reported age started smoking among current smokers at baseline

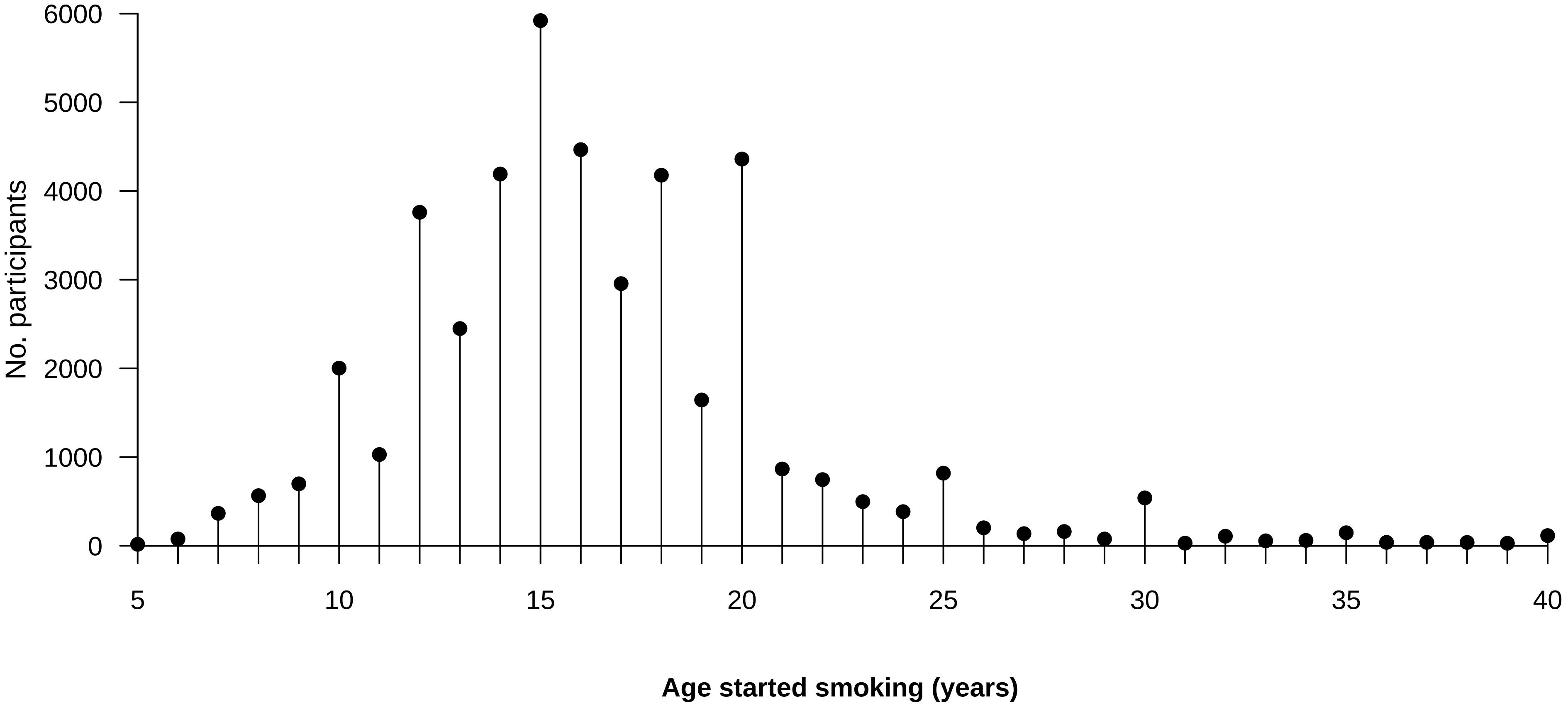

Note: the <1% of smokers who reported starting to smoke after age 40 not pictured.

Supplementary Figure 7: Cause−specific mortality at ages 30-69 years, cigarette smoker vs never-smoker, by amount smoked at baseline

Cause of death	Amount smoked*	No. deaths	RR (95% CI)
Respiratory cancers	Never	218	1.00 (0.87–1.15)
	1−9/day	105	2.74 (2.26–3.32)
	10−19/day	249	3.99 (3.53–4.52)
	20/day	290	4.81 (4.28–5.40)
	>20/day	121	6.56 (5.48–7.86)
Other cancers	Never	935	1.00 (0.93–1.07)
	1−9/day	172	1.22 (1.05–1.41)
	10−19/day	269	1.31 (1.17–1.48)
	20/day	251	1.35 (1.19–1.53)
	>20/day	97	1.78 (1.45–2.17)
COPD	Never	86	1.00 (0.80–1.25)
	1−9/day	38	2.97 (2.16–4.09)
	10−19/day	55	2.97 (2.28–3.87)
	20/day	80	4.54 (3.63–5.68)
	>20/day	30	5.92 (4.12–8.51)
Other respiratory	Never	154	1.00 (0.84–1.19)
	1−9/day	36	1.65 (1.19–2.28)
	10−19/day	47	1.40 (1.05–1.87)
	20/day	52	1.46 (1.11–1.93)
	>20/day	27	2.50 (1.70–3.67)
IHD	Never	625	1.00 (0.92–1.09)
	1−9/day	152	1.54 (1.32–1.81)
	10−19/day	285	1.83 (1.63–2.06)
	20/day	279	1.83 (1.63–2.07)
	>20/day	93	2.00 (1.63–2.45)
Stroke and other vascular	Never	618	1.00 (0.92–1.09)
	1−9/day	151	1.59 (1.36–1.86)
	10−19/day	223	1.52 (1.34–1.74)
	20/day	234	1.62 (1.42–1.85)
	>20/day	78	1.77 (1.41–2.21)
Other medical causes	Never	678	1.00 (0.92–1.09)
	1−9/day	110	1.01 (0.84–1.21)
	10−19/day	194	1.15 (1.00–1.32)
	20/day	205	1.28 (1.12–1.48)
	>20/day	68	1.41 (1.11–1.79)
External causes	Never	222	1.00 (0.87–1.16)
	1−9/day	42	0.99 (0.73–1.34)
	10−19/day	73	0.98 (0.77–1.23)
	20/day	80	1.06 (0.85–1.33)
	>20/day	36	1.53 (1.10–2.13)

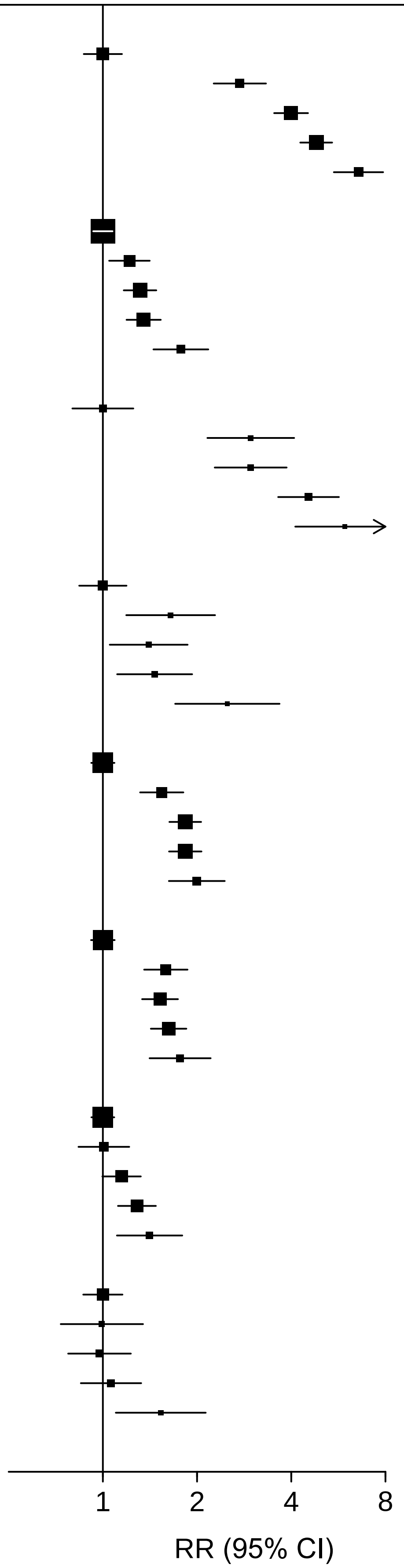

RR=mortality rate ratio, adjusted for age at risk, sex, province, education, alcohol consumption and BMI
CI=confidence interval

*Groups defined by number of cigarettes smoked reported at baseline. Mean number of cigarettes smoked at re-survey in those baseline-defined groups was 9, 14, 18, and 24 cigarettes per day, respectively.

Supplementary Figure 8: All−cause mortality at ages 30-69 years, by sex and age started smoking

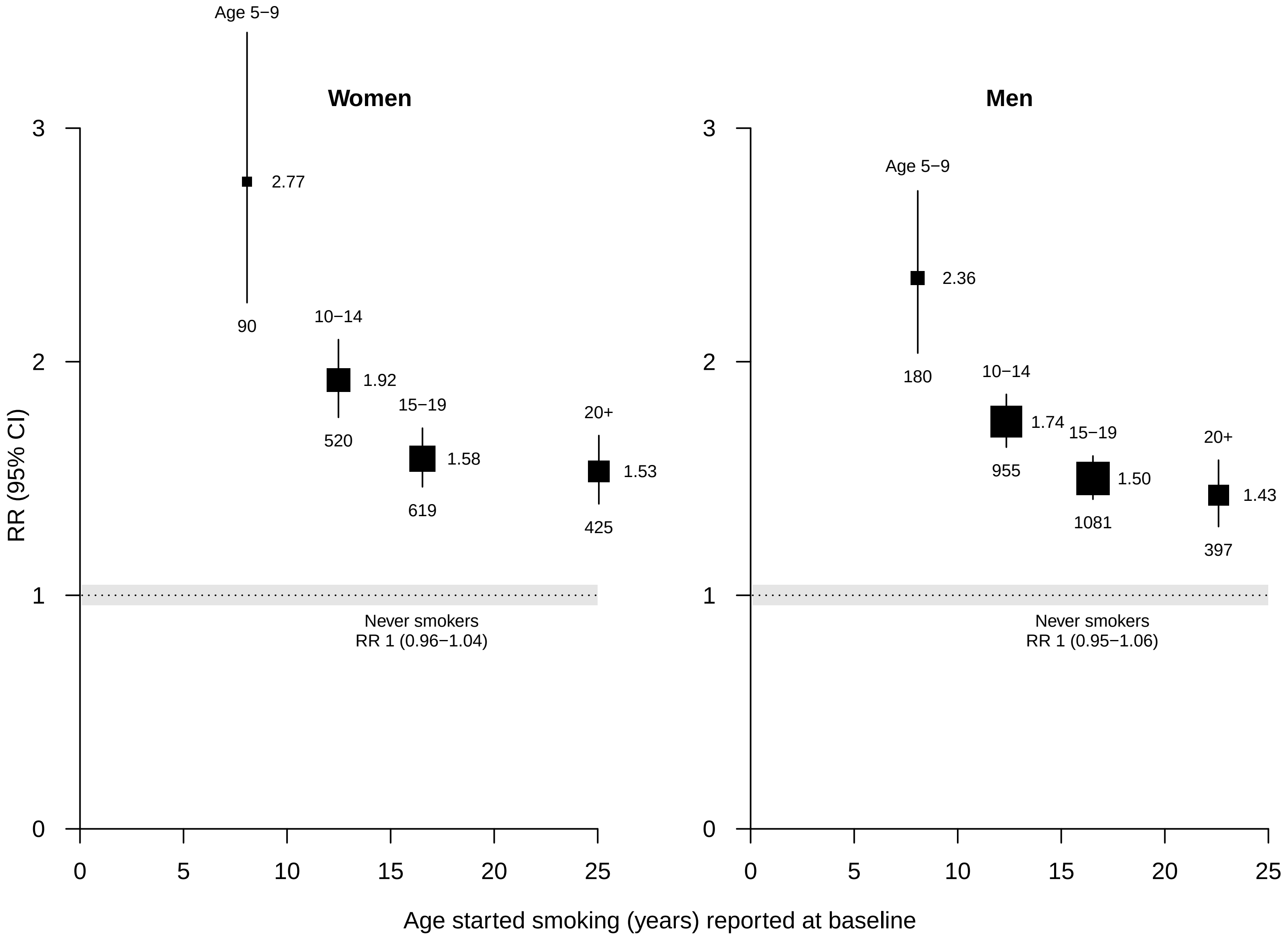

RR=mortality rate ratio adjusted for age at risk, province, education, alcohol consumption and BMI

Supplementary Figure 9: All-cause mortality at ages 30-69 years, by sex and amount smoked

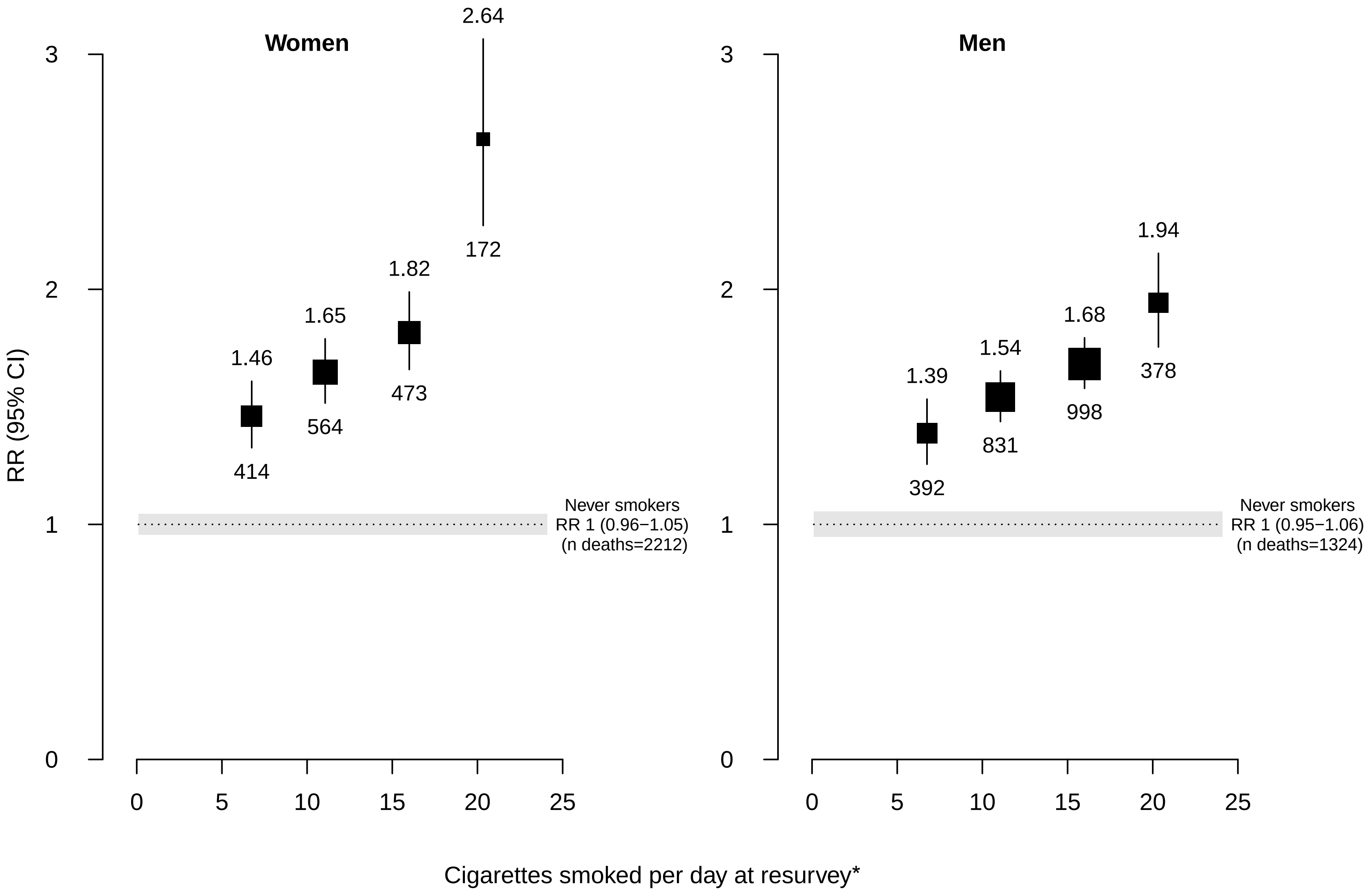

RR=mortality rate ratio adjusted for age at risk, province, education, alcohol consumption and BMI
* Mean of 6.4 years between baseline and resurvey among continuing smokers

Supplementary Figure 10: All-cause mortality at ages 30-69 years, by age started smoking and amount smoked, after mutual adjustment

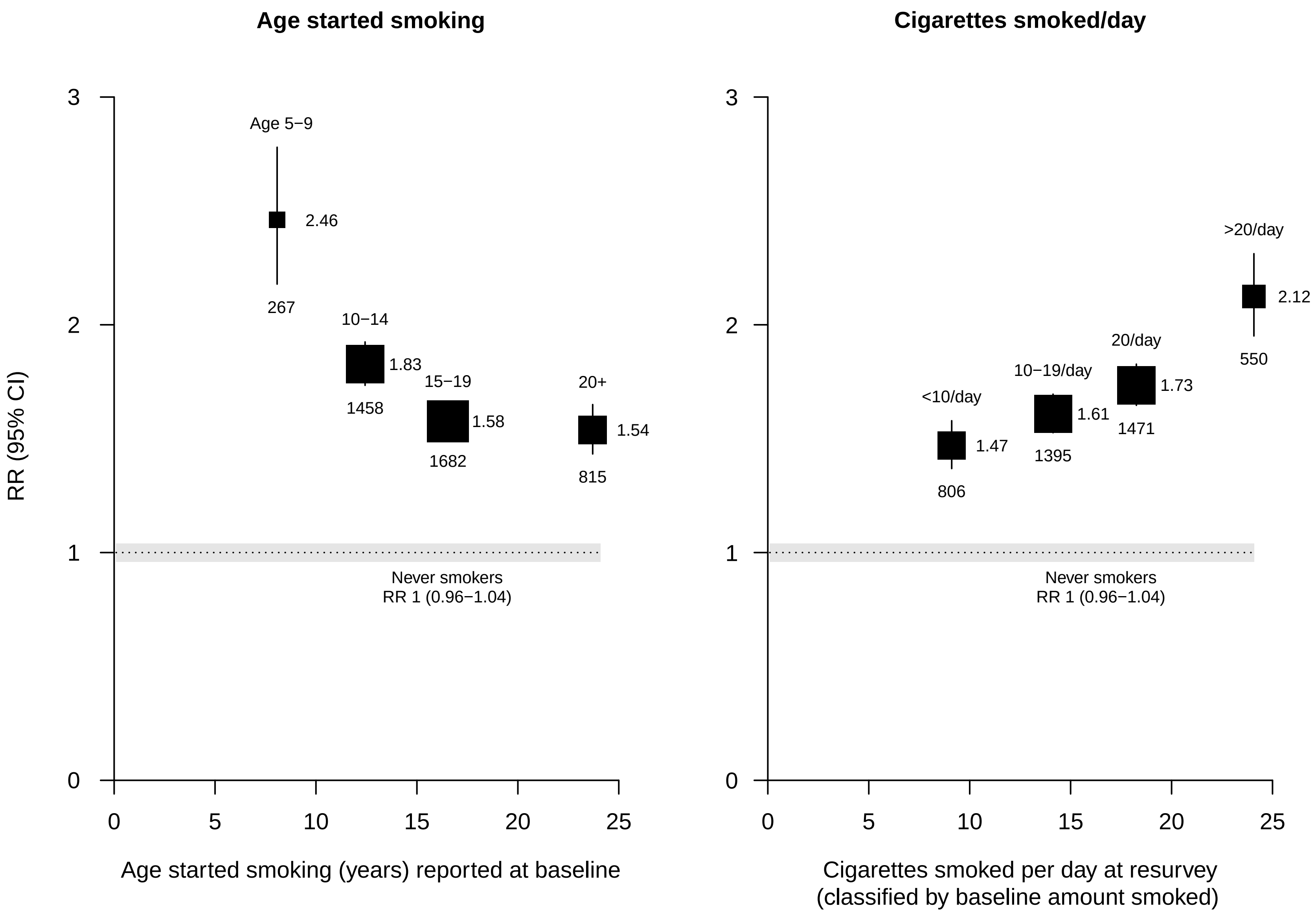

RR=mortality rate ratio adjusted for age at risk, sex, province, education, alcohol consumption and BMI

Supplementary Figure 11: All−cause mortality at ages 30−69 years, by age started smoking and amount smoked. Effect of excluding those who had quit for one or more years

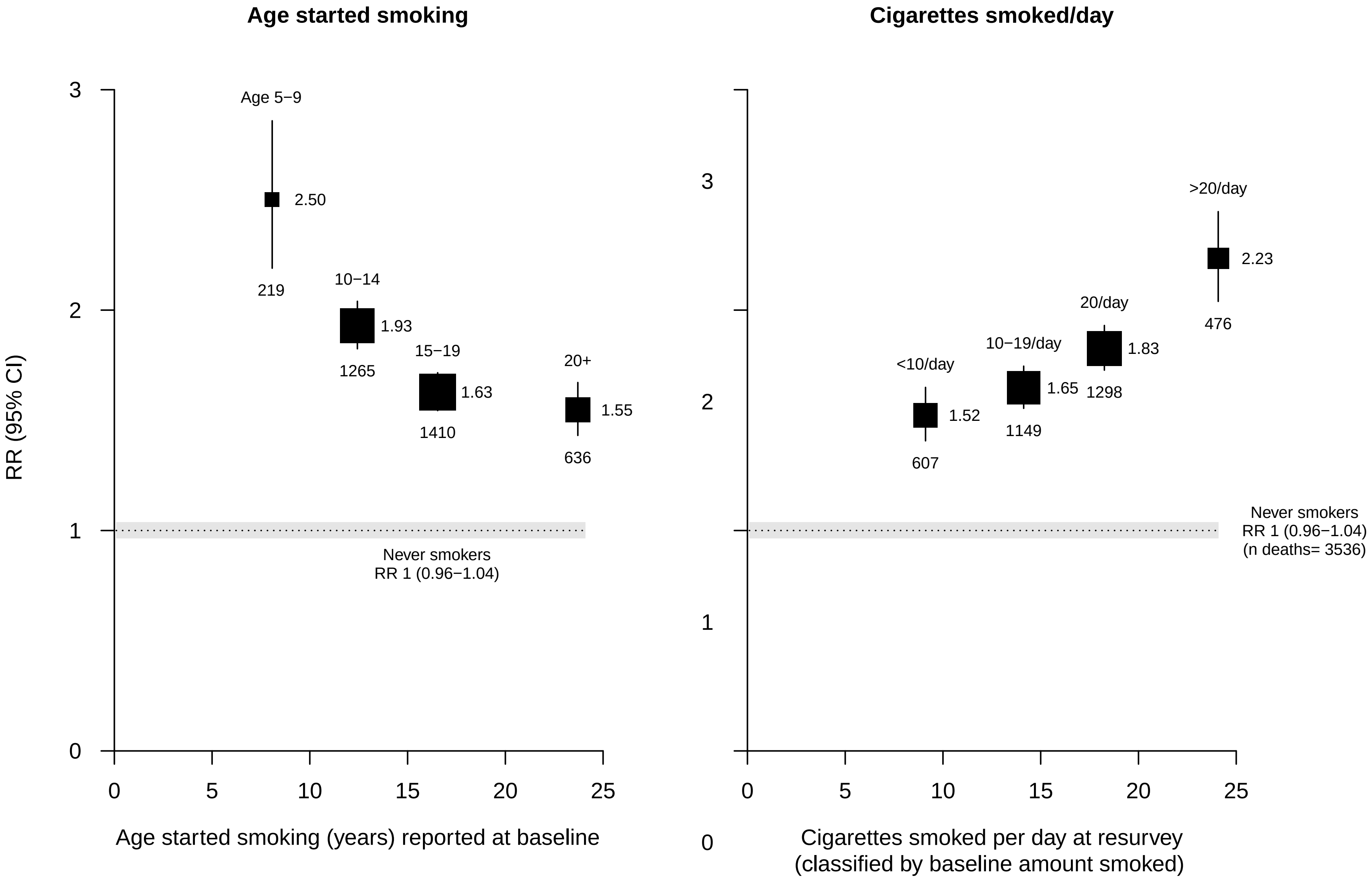

RR=mortality rate ratio adjusted for age at risk, sex, province, education, alcohol consumption and BMI

Supplementary Figure 12: All-cause mortality at ages 30-69 years for cigar only and cigarette smokers versus never-smokers

Cause of death	Baseline smoking pattern*	No. deaths	RR (95% CI)
Respiratory cancers	Never	218	1.00 (0.87–1.15)
	Cigar only	19	1.59 (1.01–2.49)
	Cigarettes	765	4.17 (3.87–4.50)
Other cancers	Never	935	1.00 (0.93–1.07)
	Cigar only	40	1.32 (0.96–1.81)
	Cigarettes	789	1.34 (1.25–1.44)
COPD	Never	86	1.00 (0.80–1.25)
	Cigar only	6	1.63 (0.73–3.67)
	Cigarettes	203	3.70 (3.21–4.26)
Other respiratory	Never	154	1.00 (0.84–1.19)
	Cigar only	9	1.27 (0.65–2.46)
	Cigarettes	162	1.59 (1.36–1.85)
IHD	Never	625	1.00 (0.92–1.09)
	Cigar only	40	1.12 (0.82–1.54)
	Cigarettes	809	1.78 (1.66–1.91)
Stroke and other vascular	Never	618	1.00 (0.92–1.09)
	Cigar only	49	1.64 (1.23–2.18)
	Cigarettes	686	1.59 (1.48–1.72)
Other medical causes	Never	678	1.00 (0.92–1.09)
	Cigar only	34	1.14 (0.81–1.60)
	Cigarettes	577	1.18 (1.09–1.28)
External causes	Never	222	1.00 (0.87–1.15)
	Cigar only	13	0.97 (0.56–1.69)
	Cigarettes	231	1.07 (0.93–1.22)
All causes	Never	3536	1.00 (0.97–1.04)
	Cigar only	210	1.27 (1.11–1.46)
	Cigarettes	4222	1.66 (1.61–1.71)

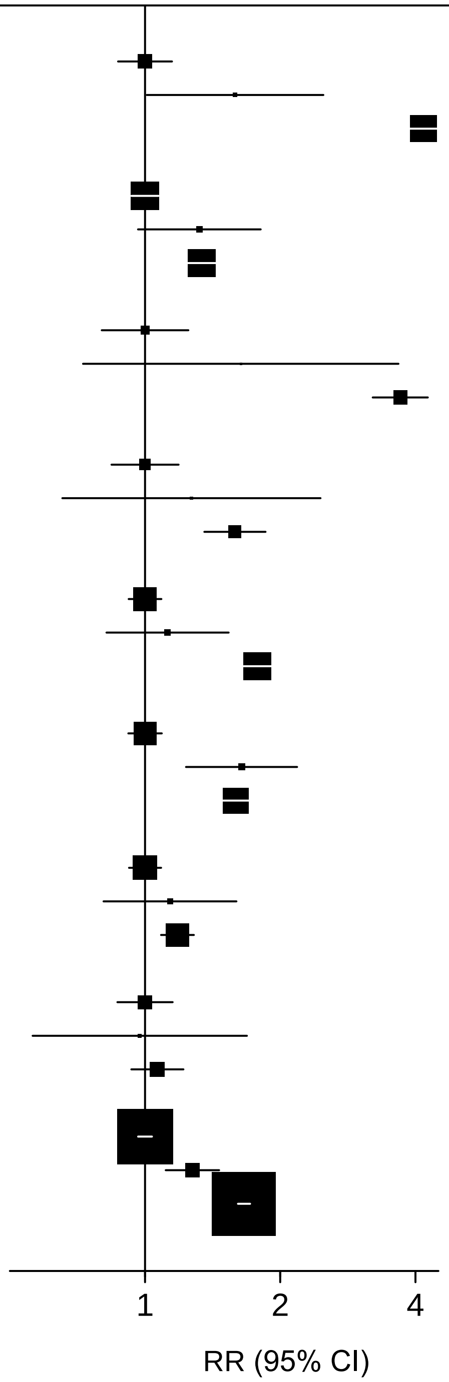

RR=mortality rate ratio adjusted for age at risk, sex, province, education, alcohol consumption and BMI
CI=Confidence interval
Cigar only: mean 3 cigars/day. Cigarette: mean 15 cigarettes/day (and 7% also smoked cigars).

Supplementary Figure 13: Mean number of cigarettes sold per adult per day in Cuba, 1980−2015

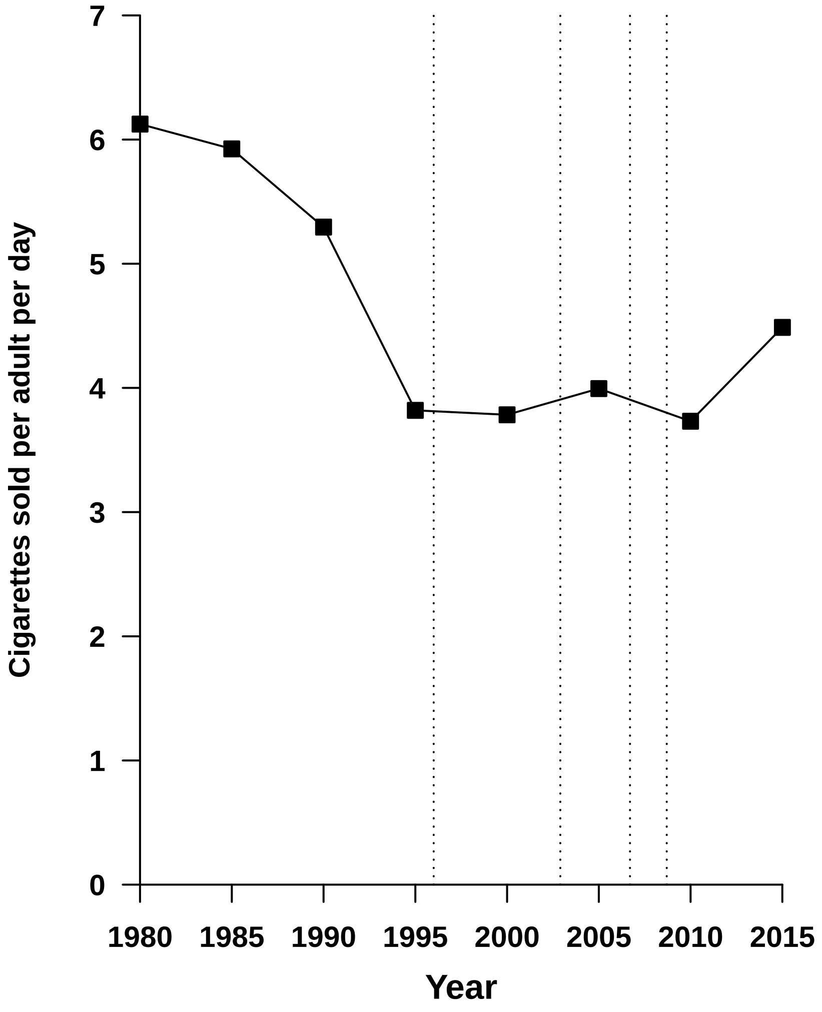

Vertical dotted lines refer to baseline survey (1996−2002) and resurvey (2006−2008) periods.

Source: Suarez−Lugo N. Consumo de cigarrillos y elasticidad precio−demanda. Cuba 2016. Horizonte Sanitario 2016; 16(3).

Supplementary Table 1: ICD-9 and ICD-10 codes for mortality endpoints

Endpoint	ICD-9 codes	ICD-10 codes
Cancer		
Lung or upper-aerodigestive	140-150, 161-162	C00-C15, C32-C34
All other	151-160, 163-208	C16-C31, C35-C97
Respiratory		
COPD	490-492, 494-496	J40-J44, J47
All other	460-489, 497-519, 010-012	A15-16, J00-J39, J48-J98
Vascular		
Ischaemic heart disease	410-414	I20-I25
Stroke and all other	390-409, 415-459, 798	I00-I19, I26-I99
External	800-1002	S00-Y89
All-cause	000-1002	A00-Y89

ICD=International Classification of Diseases; COPD=Chronic Obstructive Pulmonary Disease

Supplementary Table 2: Chacteristics of the 52 524 male participants included in the mortality analyses, by smoking pattern and age started smoking

	Never smoker	Ex smoker	Smoke cigars only	Cigarette smoker, by age started: Any	5-9	10-14	15-19	20+
**Participants**	21 515	3745	2184	25 080	1129	8210	11 323	4418
**Age, years**	50 (10)	53 (10)	57 (9)	50 (9)	52 (10)	51 (10)	49 (9)	49 (9)
**Smoking behavior**								
Age started smoking (years)	-	16.3 (4.9)	16.7 (6.8)	15.8 (4.4)	8.1 (0.9)	12.4 (1.4)	16.5 (1.3)	22.5 (4.5)
Cigar smokers	-	-	2184 (100%)	2662 (11%)	181 (16%)	1032 (13%)	1064 (9%)	385 (9%)
Cigarettes/day at baseline	-	-	-	16.2 (8.7)	19.6 (10.9)	17.5 (8.9)	15.8 (8.2)	13.7 (8.0)
Cigars/day at baseline	-	-	3.2 (3.0)	3.4 (4.0)	2.9 (3.2)	3.2 (3.7)	3.8 (4.5)	3.2 (3.6)
Current smokers at resurvey*	7%	26%	83%	85%	86%	82%	87%	85%
Cigarettes/day at resurvey*	-	-	-	17.8 (10.1)	19.7 (9.7)	19.9 (10.5)	17.3 (10.1)	14.6 (8.7)
Cigarettes/day at resurvey among all resurveyed*	0.9 (4.3)	4.6 (10.6)	2.6 (6.1)	14.6 (11.4)	15.7 (11.7)	15.7 (12.4)	14.6 (11.2)	11.9 (9.7)
**Other characteristics**								
Completed high school	11 411 (53%)	1786 (48%)	566 (26%)	11 377 (45%)	286 (25%)	3110 (38%)	5561 (49%)	2420 (55%)
Consume alcohol weekly	4376 (20%)	1011 (27%)	619 (28%)	10 354 (41%)	428 (38%)	3356 (41%)	4791 (42%)	1779 (40%)
BMI kg/m^2	24.4 (3.8)	24.5 (3.7)	24.7 (4.1)	23.8 (3.6)	23.7 (4.0)	23.7 (3.7)	23.8 (3.5)	24.0 (3.6)
SBP (mmHg)	125 (13)	127 (15)	128 (16)	125 (14)	125 (16)	124 (14)	125 (13)	125 (14)

N (%) or mean (SD) shown.
BMI=body-mass index; SBP=systolic blood pressure
* Of the 3578 participants resurveyed, 1519 were men. Among men at baseline, 732 participants reported never smoking, 57 ex-smoking, 69 cigar smoking only, and 661 cigarette smoking (mean 18/day).

Supplementary Table 3: Characteristics of the 66 316 female participantsincluded in the mortality analyses, by smoking pattern and age started smoking

	Never smoker	Ex smoker	Smoke cigars only	Cigarette smoker, by age started				
				Any	5-9	10-14	15-19	20+
**Participants**	43 671	3332	351	18 962	595	5222	7842	5303
**Age, years**	51 (10)	52 (10)	57 (9)	48 (9)	50 (10)	48 (9)	47 (9)	49 (9)
**Smoking behavior**								
Age started smoking (years)	-	18.8 (7.3)	19.5 (9.9)	17.5 (6.2)	8.1 (1.0)	12.5 (1.3)	16.5 (1.4)	24.7 (6.7)
Cigar smokers	-	-	351 (100%)	502 (3%)	26 (4%)	147 (3%)	192 (2%)	137 (3%)
Cigarettes/day at baseline	-	-	-	13.4 (8.2)	16.1 (9.6)	15.1 (8.5)	13.3 (7.8)	11.5 (7.8)
Cigars/day at baseline	-	-	3.4 (3.7)	5.6 (6.2)	2.0 (1.3)	5.1 (5.7)	7.2 (7.2)	4.7 (5.4)
Current smokers at resurvey*	3%	26%	50%	81%	86%	83%	83%	76%
Cigarettes/day at resurvey*	-	-	-	13.7 (8.4)	17.8 (9.9)	14.5 (8.0)	13.8 (7.6)	12.1 (9.4)
Cigarettes/day at resurvey among all resurveyed*	0.3 (2.3)	3.1 (6.5)	5.0 (12.2)	11.0 (9.3)	15.4 (11.1)	12.1 (9.1)	11.3 (8.7)	9.0 (9.7)
**Other characteristics**								
Completed high school	17 072 (39%)	1221 (37%)	63 (18%)	7270 (38%)	92 (15%)	1554 (30%)	3289 (42%)	2335 (44%)
Consume alcohol weekly	1235 (3%)	190 (6%)	51 (15%)	1752 (9%)	50 (8%)	553 (11%)	768 (10%)	381 (7%)
BMI kg/m²	24.8 (4.4)	24.7 (4.8)	24.7 (4.5)	23.9 (4.4)	23.6 (5.2)	23.7 (4.6)	23.8 (4.2)	24.2 (4.4)
SBP (mmHg)	124 (16)	124 (17)	128 (17)	122 (16)	123 (19)	121 (16)	122 (16)	122 (16)

N (%) or mean (SD) shown.
BMI=body-mass index; SBP=systolic blood pressure
* Of the 3578 participants resurveyed, 2059 were women. Among women at baseline, 1501 participants reported never smoking, 34 ex-smoking, 6 cigar smoking only, and 518 cigarette smoking (mean 13/day).

Supplementary Table 4: Characteristics at resurvey about 6 years later, by baseline smoking status and amount smoked

	Never smoker	Ex smoker	Smoke cigars only	Cigarette smoker, by cigarettes/day at recruitment				
				Any	< 10	10-19	20	21+
**Participants**	2243	92	76	1186	262	350	430	144
**Smoking status at resurvey**								
Never-smoker	2070 (92%)	0 (0%)	0 (0%)	1 (<0.5%)	0 (0%)	0 (0%)	0 (0%)	1 (1%)
Ex-smoker	73 (3%)	68 (74%)	15 (20%)	194 (16%)	63 (24%)	67 (19%)	50 (12%)	14 (10%)
Smoke < 20 cigs/day	62 (3%)	13 (14%)	10 (13%)	525 (44%)	168 (64%)	199 (57%)	126 (29%)	32 (22%)
Smoke 20 cigs/day	23 (1%)	5 (5%)	4 (5%)	317 (27%)	19 (7%)	57 (16%)	198 (46%)	43 (30%)
Smoke >20 cigs/day	6 (<0.5%)	4 (4%)	2 (3%)	121 (10%)	5 (2%)	18 (5%)	49 (11%)	49 (34%)
Smoke cigars only	9 (<0.5%)	2 (2%)	45 (59%)	28 (2%)	7 (3%)	9 (3%)	7 (2%)	5 (3%)
**Average daily cigs**								
At baseline	-	-	-	15.8 (9.5)	4.8 (2.0)	11.5 (2.2)	20.0 (0.0)	34.0 (8.6)
At resurvey, per smoker *	12.3 (9.6)	16.8 (11.9)	13.0 (8.9)	16.0 (9.6)	9.3 (7.5)	14.1 (7.6)	18.3 (7.5)	24.1 (13.0)
At resurvey, all participants †	0.5 (3.1)	4.0 (9.2)	2.7 (6.6)	13.0 (10.7)	6.8 (7.6)	11.1 (8.9)	15.8 (9.4)	20.7 (14.7)
**Average daily cigars**								
At baseline	-	-	2.4 (1.4)	0.1 (0.4)	0.0 (0.2)	0.1 (0.4)	0.1 (0.4)	0.1 (0.3)
At resurvey, per smoker *	0.3 (1.1)	0.3 (0.7)	1.9 (1.7)	0.1 (0.6)	0.2 (0.7)	0.1 (0.7)	0.1 (0.6)	0.1 (0.6)
At resurvey, all participants †	0.0 (0.2)	0.1 (0.4)	1.5 (1.7)	0.1 (0.6)	0.1 (0.6)	0.1 (0.6)	0.1 (0.6)	0.1 (0.5)

N (%) or mean (SD) shown. Includes only participants with non-missing smoking information, and non-missing blood pressure and BMI values
* Among those self-reporting current smoking at resurvey
† Among all participants, ie, those reporting never- or ex-smoking assigned zero cigarettes/day

Supplementary Table 5: Cause-specific population-attributable fractions (PAF) at ages 30-69 years, by sex

	Male			Female		
**Cause of death**	**No. of deaths smokers/non-smokers**	**RR (95% CI)**	**PAF (%)**	**No. of deaths smokers/non-smokers**	**RR (95% CI)**	**PAF (%)**
IHD	662/304	1.55 (1.35-1.78)	24	320/321	2.03 (1.74-2.38)	25
Stroke and other vascular	526/223	1.57 (1.34-1.84)	26	321/395	1.60 (1.38-1.86)	17
Respiratory cancers	575/104	3.22 (2.60-3.99)	58	271/114	4.61 (3.69-5.75)	55
Other cancers	473/236	1.30 (1.11-1.53)	15	478/699	1.34 (1.19-1.51)	10
COPD	115/23	3.25 (2.06-5.12)	58	113/63	3.48 (2.54-4.77)	46
Other respiratory	110/62	1.21 (0.88-1.66)	11	78/92	1.80 (1.33-2.45)	20
Other medical	441/230	1.25 (1.06-1.47)	13	276/448	1.19 (1.02-1.38)	6
All medical	2902/1182	1.61 (1.50-1.73)	27	1857/2132	1.72 (1.61-1.83)	19

IHD=Ischaemic Heart Disease; COPD=Chronic Obstructive Pulmonary Disease

Supplementary Table 6: Comparisons between the never-smoker lung cancer mortality rates in the Cuba Prospective Study during 1996-2016 and the American Cancer Society prospective study during 1984-88

Age at risk (years)	Person years	Cuba rate/100000	ACS 1984-88 rate/100000	Cuba study lung ca. deaths	Expected deaths at ACS rate
**Men**					
40-44	31886	0	3	0	1
45-49	47970	2.1	5	1	2
50-54	59413	10.1	7	6	4
55-59	57494	19.1	10	11	6
60-64	53857	53.8	14	29	8
65-69	47224	57.2	20	27	9
70-74	39586	98.5	27	39	11
75-79	31523	92.0	35	29	11
**Women**					
Age at risk (years)	Person years	Cuba rate/100000	ACS 1984-88 rate/100000	Cuba study lung ca. deaths	Expected deaths at ACS rate
40-44	60140	3.3	3	2	2
45-49	91629	2.2	4	2	4
50-54	116435	7.7	7	9	8
55-59	118020	13.6	10	16	12
60-64	113870	28.1	14	32	16
65-69	105126	39.0	19	41	20
70-74	92355	40.1	26	37	24
75-79	75781	42.2	34	32	26

Source: appendix to Lancet 1992; 339: 1268-78

Supplementary Table 7: Baseline characteristics of 1997-2014 US National Health Interview Surveys

	Never smoker	Ex-smoker	Daily* smoker	Daily smokers, by age began regular smoking					Total
				< 10	10-14	15-17	18-20	> 20	
	n=186 040	n=77 315	n=63 101	n=1225	n=12 111	n=21 381	n=16 132	n=12 252	n=326 456
**Mean (SD):**									
Age started smoking†	-	18 (4)	18 (5)	8 (1)	13 (1)	16 (1)	19 (0.1)	26 (5)	-
Cigarettes/day	-	-	17 (10)	21 (13)	20 (11)	18 (10)	16 (9)	14 (9)	-
Age (in range 30-69)	46 (11)	52 (11)	47 (10)	49 (11)	47 (10)	46 (10)	47 (10)	48 (10)	48 (11)
**Characteristics (% of n):**									
Male	40%	53%	50%	68%	55%	50%	49%	44%	45%
Consume alcohol weekly	25%	37%	37%	36%	37%	38%	38%	34%	30%
High school graduate	85%	86%	78%	58%	70%	76%	84%	84%	84%
Race/ethnicity, self-defined									
Non-Hispanic White	57%	73%	71%	72%	75%	74%	70%	62%	64%
Non-Hispanic Black	16%	10%	15%	12%	11%	14%	16%	21%	14%
Hispanic	21%	12%	10%	11%	10%	9%	10%	12%	17%
Other	7%	4%	4%	5%	4%	4%	5%	5%	6%
Region									
North-East	17%	19%	17%	14%	18%	18%	17%	15%	18%
Mid-West	21%	23%	26%	24%	25%	26%	27%	24%	22%
South	37%	34%	39%	41%	38%	39%	38%	41%	37%
West	25%	24%	18%	20%	19%	17%	18%	20%	23%

Analyses restricted to 326 456 adults surveyed at ages 30-69 in the 1997-2014 annual National Health Interview Surveys (NHIS).
*Smokers who at the time they were surveyed did not smoke daily are excluded from this table.
†The few who reported starting regular smoking before age 6 were assigned age 6 during data collection.

ESTUDIO CUBANO SOBRE TABACO Y SALUD

Provincia No. ☐ 1-9 Municipio No. ☐☐ 0-99 Area de Salud No. ☐ 0-9

№ 17524 A

Carnet de Identidad No. |_|_|_|_|_|_|–|_|_|_|_|_| Fecha de nacimiento (a.m.d) + 5 números
(11 números: excluir si no tiene C.I. de 6 + 5 números)

Nombre de la persona y dirección: |_|_|_|_|_|_|_|_|_|_|_|_|_|_|_|_|_|_|_|_|_|_|_|_|_|_|_|_|_|_|
Calle No. Teléfono

Nombre del familiar y dirección: |_|_|_|_|_|_|_|_|_|_|_|_|_|_|_|_|_|_|_|_|_|_|_|_|_|_|_|_|_|_|
Calle No. Municipio Provincia Teléfono

LETRA DE MOLDE, POR FAVOR

Sexo (1 = masc, 2 = fem.) ☐ 1-2

Color de piel (1 = blanco, 2 = negro, 3 = mestizo, 4 = otra) ☐ 1-4

Estado social (1 = soltero/viudo, 2 = casado) ☐ 1-2

Educación completada (0 = ninguno*, 1 = primaria, 2 = secundaria, 3 = técnico medio, 4 = obrero calificado, 5 = pre-universitario, 6 = universitario) ☐ 0-6

Categoría ocupacional (0 = ninguno*, 1 = dirigente, 2 = profesional, 3 = técnico, 4 = de servicio, 5 = agrícola, 6 = industria, 7 = militar) ☐ 0-7

* Si es cero (Ej. ama de casa, jubilado o desempleado), diga si ha trabajado alguna vez. ☐ 0-7

Quisiera medir su presión arterial mientras esta sentado, hacerle unas pocas preguntas sobre tabaco y alcohol, y medir su PA de nuevo.

Presión arterial (una sola toma, sentado): |_|_|_| / |_|_|_| PAS / PAD

Ahora, TABACO: ¿Ha fumado alguna vez regularmente (Ej. en la mayoría de los días)?
SI NO, vaya directamente a las preguntas sobre ALCOHOL. ☐ S=Sí, N=No

¿A QUE EDAD APROXIMADAMENTE COMENZO USTED A FUMAR REGULARMENTE? ☐☐ años de edad

¿Desde entonces ha dejado de fumar alguna vez por un año o más? ☐ S=Sí, N=No

¿Aproximadamente cuántos cigarrillos por día se fumó usted el último mes? (0 = ninguno) ☐☐ diariamente

¿Aproximadamente cuántos tabacos por día se fumó usted el último mes? (0 = ninguno) ☐☐ diariamente

¿Si no fumó el último mes, a que edad dejó usted de fumar? (en blanco si no es aplicable) ☐☐ años de edad

Ahora me gustaría hacerle algunas preguntas sobre ALCOHOL:

¿Aproximadamente cuántos días a la semana toma usted ron u otra bebida fuerte? (0 = cero días o usualmente menos de una vez por semana) ☐ 0-7

¿El día que usted toma ron (u otra bebida fuerte) cuántas líneas se toma? ☐☐ líneas

¿Aproximadamente cuántos días a la semana toma usted cerveza? (0 = cero días o usualmente menos de una vez por semana) ☐ 0-7

¿El día que usted toma cerveza, cuántas botellas se toma (aprox.) ☐☐ botellas

Finalmente, algunos aspectos médicos: Quisiera preguntarle sobre algunas enfermedades. Lo que quiero conocer es si usted alguna vez ha sido tratado por alguna de estas enfermedades. Si no lo ha sido, conteste No (N).

Asma	☐ S/N	A.V.E. (definitivo/probable)	☐ S/N	Cirrosis	☐ S/N
Enfisema (E.P.O.C.)	☐ S/N	Infarto miocardio "	☐ S/N	Enf. renal crónica	☐ S/N
Diabetes	☐ S/N	Angina pectoris "	☐ S/N	Cáncer de piel	☐ S/N
Ulcera péptica	☐ S/N	Hipertensión arterial	☐ S/N	Otro cáncer*	☐ S/N

¿*Si Otro Cáncer = Sí, dónde le comenzó el cáncer? ☐ 0-9

(1 = boca o faringe, 2 = laringe, 3 = pulmón, 4 = esófago, 5 = estómago, 6 = páncreas, 7 = cuello útero, 8 = vejiga, 9 = mama, 0 = otro, más de un sitio, o no conocido)

¿Esta usted tomando alguna medicina regularmente desde hace más de un mes? Si es Sí: ¿Qué medicinas esta tomando? (Marque con X)

Digoxina	☐	Nitratos	☐	Hipoglic. oral	☐	Anticoncept.	☐	Ca. Antag.	☐
Beta B.	☐	Inh. ACE	☐	Insulina	☐	Antidepres.	☐	Broncodilat.	☐
Diurético	☐	ASA	☐	Esteroides	☐	Ansiolítico	☐	Antiácido/H2	☐

MUCHAS GRACIAS POR SUS RESPUESTAS: Ahora necesito tomarle de nuevo su presión arterial. Repita la toma de PA (una sola toma sentado): |_|_|_| / |_|_|_| PAS/PAD

¿Cuál es la última cifra PA que tiene en su historia clínica antes de hoy?

Fecha de la última PA (a.m.d) |_|_|_|_|_|_| & |_|_|_| / |_|_|_| PAS/PAD

¿Cuál es su *TALLA* aproximada? (medirlo o preguntarlo) |_|_|_| cm

¿Cuál es su *PESO* aproximado? (pesarlo o preguntarlo) |_|_|_| kg

Fecha de la entrevista |_|_|_|_|_|_| (a.m.d)

Por favor chequee TODA la encuesta ¡MUCHAS GRACIAS!

Calidad de la cooperación (1 = pobre, 2 = regular, 3 = buena) ☐ 1-3

Nombre y apellidos del entrevistador: |_|_|_|_|_|_|_|_|_|_|_|_|_|_|_|_|_|_|_|_|_|_|_|_|_|_|

¿Algún comentario? ....

PARA LA SIGUIENTE PERSONA, UTILICE EL REVERSO DE ESTA PAGINA →

CUBAN STUDY OF TOBACCO AND HEALTH: PROSPECTIVE

Cuba Prospective Study

Province Nº ☐ 1-9 Municipality Nº ☐☐ 0-99 Health Area Nº ☐ 0-9 Identity Card Nº ☐☐☐☐☐☐-☐☐☐☐☐ Date of birth (y,m,d)

Name of interviewee: ____________________

Address: ____________________
Street Nº Municipality Province Telephone

Name of friend: ____________________

Address: ____________________
Street Nº Municipality Province Telephone

CAPITAL LETTERS, PLEASE!

Sex (1=masc, 2=fem) ☐ 1-2

Skin colour (1=white, 2=black, 3=mixed, 4=other) ☐ 1-4

Marital status (1=single/widowed, 2=married) ☐ 1-2

Education completed (0=none, 1=primary, 2=secondary, 3=technician, 4=skilled worker, 5=high school, 6=university) ☐ 0-6

Occupational category (0=none, 1=manager, 2=professional, 3=technician, 4=service worker, 5=agricultural, 6=industrial, 7=military; if housewife, retired or unemployed, we would like to know if you were employed in the past) ☐ 0-7

(☐ 0-7)

What I'd like to do is measure your blood pressure while you are sitting down, ask you a few questions about tobacco and alcohol, and then do your blood pressure again. Blood pressure (single measurement, seated): ☐☐☐ / ☐☐☐ SBP / DBP

Now, TOBACCO: Have you EVER smoked regularly (i.e. on most days for at least a year)? If NO, then go directly to the ALCOHOL questions ☐ **Y=Yes, N=No**

AT ABOUT WHAT AGE DID YOU FIRST START SMOKING TOBACCO REGULARLY? ☐☐ years of age

Since then, have you ever STOPPED smoking for a year or more? ☐ Y=Yes, N=No

About how many cigarettes a day did you smoke last month? (0=none) ☐☐ daily

About how many cigars a day did you smoke last month? (0=none) ☐☐ daily

If NO SMOKING last month, at about what age did you last stop smoking? (blank = not applicable) ☐☐ years of age

Now I'd like to ask you some questions about ALCOHOL:

About how many days a week would you drink any rum or other spirits? (0=none or usually less than once a week) ☐ 0-7

On a day when you drink rum (or other spirits), about how many glasses ("lineas") would you drink? ☐☐ lineas

About how many days a week would you drink beer? (0=none or usually less than once a week) ☐ 0-7

On a day when you do drink beer, about how many bottles would you drink? ☐☐ bottles

Finally, MEDICAL MATTERS: I'd like to ask about various different illnesses. What I want to know for each illness is whether you've ever had medical attention for it. If you've never heard of the illness, then just say NO.

Asthma	☐ Y/N	Stroke (definite/probable)	☐ Y/N	Cirrhosis of the liver	☐ Y/N
Emphysema/C.O.P.D.	☐ Y/N	Myocardial infarction "	☐ Y/N	Chronic kidney disease	☐ Y/N
Diabetes	☐ Y/N	Angina pectoris "	☐ Y/N	Skin cancer	☐ Y/N
Peptic ulcer	☐ Y/N	Hypertension	☐ Y/N	Other cancer*	☐ Y/N

*If Other cancer, where in your body did the cancer start? ☐ 0-9
(1=mouth, 2=larynx, 3=lung, 4=oesphagus, 5=stomach, 6=pancreas, 7=cervix, 8=bladder, 9=breast, 0=other, more than 1 site, or not known)

Have you been taking any regular medication for more than 1 month? If Yes: What such medicines are you now taking? (Mark with an X)

Digoxin ☐	Nitrates ☐	Oral hypoglycaemic ☐	O.Contraceptive ☐	Ca. antagonist ☐
Beta blocker ☐	ACE inhibitor ☐	Insulin ☐	Antidepressant ☐	Bronchodilator ☐
Diuretic ☐	Aspirin ☐	Steroids ☐	Tranquillisers ☐	Antacid/H2 ☐

THANK YOU VERY MUCH FOR YOUR ANSWERS. Now we just need one more blood pressure. Repeat blood pressure (single measurement, seated) ☐☐☐ / ☐☐☐ PAS / PAD

What was the last blood pressure in the patient's medical record **before** today?

Date of last BP (y,m,d) ☐☐☐☐☐☐ & ☐☐☐ / ☐☐☐ PAS / PAD

What is your approximate **HEIGHT** (either ask or measure)? ☐☐☐ cm

What is your approximate **WEIGHT** (either ask or measure)? ☐☐☐ kg

Date of interview ☐☐☐☐☐☐ (y,m.d)

Quality of co-operation (1=poor, 2=fair, 3=good) ☐ 1-3

Please check that **ALL** the questions have been answered, so that there is no need to write back to you for extra details.
MANY THANKS!

Name of interviewer: please PRINT ____________________

Any comments: ....

Brief report on analyses of the 1997-2014 US National Health Interview Surveys

Introduction: We investigate the association between age started smoking and adult mortality, using public data from the annual US National Health Interview Surveys (NHIS).[1]

Methods: 552 837 US adults aged ≥18 years were surveyed in 1997-2014 and followed until 1.1.2016 via the National Death Index. They were asked whether they smoked, how much they smoked, and when they had started regular smoking. The few who smoked, but not every day, were excluded. Current smokers were grouped by the age when they had begun daily smoking (<10, 10-14, 15-17, 18-20, or >20 years). Adjusted Cox regression was used to estimate smoker versus never-smoker mortality rate ratios at ages 30-69 years in each group. Rate ratios (with group-specific 95% CIs[2]) were adjusted for age-at-risk (8 categories), sex, race (4 categories), education (4 categories), region (4 categories), and drinking (5 categories). NHIS surveys were conducted by the National Center for Health Statistics; participants gave informed consent. We used SASv9.4 (SAS Institute) for statistical analyses, and Rv3.1.1 for graph-plotting.

Results: Among 326 456 adults aged 30-69 without missing demographic or mortality information, 63 101 (19%) were daily smokers, 77 315 (24%) ex-smokers, and 186 040 (57%) never-smokers. Of daily smokers, 13 336 (21%) had started smoking regularly in childhood (ie, before age 15), of whom 1225 (2%) had started before age 10 (Table). Compared with those who had started later, those who had started in childhood were more likely to be male, more likely to be non-Hispanic white, less likely to graduate high school, equally likely to drink, but smoked more cigarettes/day.

During 2.2 million person-years of follow-up at ages 30-69, there were 6698 deaths among never-smokers and 6691 among daily smokers. The fully adjusted all-cause mortality rate ratio comparing daily versus never-smokers was 3.70 (95% CI 3.25-4.21) for those who had started before age 10, 2.91 (2.76-3.06) for those who had started at ages 10-14, 2.43 (2.33-2.54) for those who had started at ages 15-17, 2.20 (2.09-2.32) for those who had started at ages 18-20, and 2.04 (1.93-2.17) for those who had started after age 20. These associations were little altered by further adjustment for cigarettes/day.

Discussion: For those who had started after age 20, current smoking approximately *doubled* all-cause mortality rates at ages 30-69, but for those who had started in childhood current smoking approximately *tripled* all-cause mortality rates at ages 30-69, with the highest rates in the few who had started before age 10. According to the 2017 NHIS, about 5 million of the 25 million daily

smokers in the US had started in childhood, among whom 0.5 million had started before age 10. These 5 million smokers will be at particularly high risk of death from smoking if they do not quit.

Tobacco surveillance systems routinely report the incidence and prevalence of smoking before age 15 in the United States[3] and other populations,[4] and those who start smoking before age 10 will, if they continue, be at even greater risk of premature death than children who start at 10-14. Starting daily smoking in childhood approximately triples the risk of premature death at ages 30-69, and if this three-fold death rate ratio continues at older ages among those who continue to smoke then it means that about two-thirds of those who start smoking before 15 and do not stop will eventually be killed by their habit. These US findings (which match similar findings from Cuba in this report) mean that adult smokers who started in childhood or adolescence can expect particularly large gains from smoking cessation.

References

1. Centers for Disease Control and Prevention. National Health Interview Survey. https://www.cdc.gov/nchs/nhis/nhis_questionnaires.htm. Accessed February 27, 2019.

2. Plummer M. Improved estimates of floating absolute risk. *Stat Med.* 2004;23(1):93-104.

3. Jamal A, Gentzke A, Hu SS, et al. Tobacco Use Among Middle and High School Students - United States, 2011-2016. *MMWR Morb Mortal Wkly Rep.* 2017;66(23):597-603.

4. Warren CW, Lea V, Lee J, Jones NR, Asma S, McKenna M. Change in tobacco use among 13-15 year olds between 1999 and 2008: findings from the Global Youth Tobacco Survey. *Glob Health Promot.* 2009;16(2 Suppl):38-90.
